# A conserved rhizobial peptidase that interacts with host-derived symbiotic peptides

**DOI:** 10.1038/s41598-021-91394-x

**Published:** 2021-06-03

**Authors:** Alex B. Benedict, Prithwi Ghosh, Samuel M. Scott, Joel S. Griffitts

**Affiliations:** Department of Microbiology and Molecular Biology, Brigham Young University, Provo, UT 84602 USA

**Keywords:** Peptides, Bacterial genetics, Bacterial host response, Plant symbiosis, Symbiosis, Proteolysis, Proteases

## Abstract

In the *Medicago truncatula-Sinorhizobium meliloti* symbiosis, chemical signaling initiates rhizobial infection of root nodule tissue, where a large portion of the bacteria are endocytosed into root nodule cells to function in nitrogen-fixing organelles. These intracellular bacteria are subjected to an arsenal of plant-derived nodule-specific cysteine-rich (NCR) peptides, which induce the physiological changes that accompany nitrogen fixation. NCR peptides drive these intracellular bacteria toward terminal differentiation. The bacterial peptidase HrrP was previously shown to degrade host-derived NCR peptides and give the bacterial symbionts greater fitness at the expense of host fitness. The *hrrP* gene is found in roughly 10% of *Sinorhizobium* isolates, as it is carried on an accessory plasmid. The objective of the present study is to identify peptidase genes in the core genome of *S. meliloti* that modulate symbiotic outcome in a manner similar to the accessory *hrrP* gene. In an overexpression screen of annotated peptidase genes, we identified one such symbiosis-associated peptidase (*sap*) gene, *sapA* (SMc00451). When overexpressed, *sapA* leads to a significant decrease in plant fitness. Its promoter is active in root nodules, with only weak expression evident under free-living conditions. The SapA enzyme can degrade a broad range of NCR peptides in vitro.

## Introduction

The symbiosis between legumes and rhizobia is initiated by the action of a variety of plant and bacterially-derived molecules including flavonoids^1–3^, lipochitooligosaccharide nodulation (Nod) factors^4,5^, and exopolysaccharides (EPS)^6,7^. Flavonoids secreted from plant roots stimulate the production of rhizobial Nod factor, which initiates the symbiotic developmental process by causing root hairs to curl around the bacteria^8–10^. In the *Sinorhizobium meliloti* – *Medicago truncatula* symbiosis, production of the bacterial EPS succinoglycan enables the development of infection threads within root hairs, allowing the rhizobia to colonize plant tissue^11,12^. A portion of bacteria within the infection threads are endocytosed into specialized nodule cells^13,14^, forming organelle-like assemblies called symbiosomes where the intracellular rhizobia (termed bacteroids) fix atmospheric nitrogen for the plant. While interkingdom signaling molecules play critical roles during bacterial entry and early nodule development, it has also become clear that symbiotic communication is occurring around the time that nitrogen fixation commences. This is evidenced by studies in which random pairings of symbiotically competent *Medicago* hosts and *Sinorhizobium* strains often give rise to infected nodules that do not fix nitrogen^15–18^. Additionally, several Fix^−^ *M. truncatula* mutants have been isolated that allow bacterial entry into nodule cells, but nitrogen fixation is abolished^19,20^.

The first evidence of a new class of late-stage symbiotic signals emerged from a transcriptomic analysis in *M. truncatula*, where a large assortment of hundreds of host-derived nodule-specific cysteine-rich (NCR) peptides was observed^21^. Subsequent studies of NCR peptides revealed that they possess structural and bactericidal properties similar to the defensin class of antimicrobial peptides^22,23^. At non-lethal doses, NCR peptides have several effects on rhizobial cells: they permeabilize membranes, promote genome endoreduplication, and drive cell-enlargement and branching, all of which presumably facilitate effective nitrogen fixation, nutrient exchange, and suppression of rhizobial proliferation^24–29^. Genetic defects in a *Medicago* symbiosis-specific protein secretion pathway have been shown to block NCR peptide delivery to symbiosomes and prevent nitrogen fixation^26,30^. More recently, genetic disruption of specific NCR peptide-encoding genes has been linked to failure to fix nitrogen^31,32^. Taken together, legume-derived NCR peptides clearly play major roles in the later stages of symbiotic development, leading to the terminal differentiation of rhizobia within nodule cells and driving the nitrogen fixation and nutrient exchange that is characteristic of this symbiotic interaction.

The influence of host-derived NCR peptides on nodule-bound bacteria has given rise to a model in which corresponding bacterial peptidases may modulate this influence. It was previously demonstrated that a rhizobial metallopeptidase, HrrP, strongly suppresses nitrogen fixation in specific *Medicago-Sinorhizobium* combinations^33^. It was further shown that HrrP degrades NCR peptides in vitro, pointing to a direct host-strain interaction at the level of host NCR peptides and a microbial peptidase^33^. The *hrrP* gene, however, is found on a relatively rare accessory plasmid present in about 10% of *Sinorhizobium* isolates^34^. We have more recently turned attention to the question of whether NCR peptides are influenced, perhaps in more subtle ways, by peptidases that are more generally conserved in the core genome of *S. meliloti*. Considering that an ensemble of cooperating peptidases may make identification difficult by loss-of-function genetic analysis, we employed a plasmid-based overexpression screen focused on gene candidates that most likely encode peptide-hydrolyzing enzymes.

## Results

### Identification of core genome-encoded peptidase candidates

Using the online *S. meliloti* 1021 genome and *MEROPS* peptidase databases, we identified 131 putative peptidase candidates that could be screened for effects on symbiosis. An OrthoMCL analysis of the 23 fully sequenced *S. meliloti* strains in the NCBI database showed there are 70 orthologs from our set of 131 that are present in all strains^35^ (see Supplementary Table S1). The majority of the 131 putative peptidases (68%) are encoded on the main chromosome with the remaining 32% divided evenly between megaplasmids pSymA and pSymB. A similar distribution of peptidase genes is seen on the three primary replicons of the other fully sequenced strains of *S. meliloti* (data not shown). The 4 catalytic mechanisms for proteolysis represented in this set are metallopeptidases (37%), serine (28%), cysteine (7%), and aspartic (5%) proteases, with 23% having unknown or unannotated mechanisms. To predict the subcellular localization of each peptidase we used PSORTb 3.0^36^ and it was determined that nearly half (49%) are predicted to localize to the cytoplasm. Another 16% are predicted to localize to the cytoplasmic membrane, 5% to the periplasm, 1% to the outer membrane, with 3% secreted extracellularly, while 26% have no predicted localization.

### Effects of peptidase overexpression on plant fitness

Genes corresponding to 28 candidate bacterial peptidases were amplified from the *S. meliloti* 1021 genome and cloned in the overexpression vector pPG013 (Supplementary Fig. S1), which is replicated at ~ 7 copies/cell. Expression of a candidate peptidase was driven by the *hrrP* promoter (P*hrrP*) rather than its native promoter because P*hrrP* has been shown to be highly active in nodules^33^. After cloning, individual overexpression plasmids were mated into *S. meliloti* C307 for inoculating *M. truncatula* A20 plants. This particular strain-host pair was chosen because of the previous observation that expression of *hrrP* in *S. meliloti* C307 leads to a significant fitness reduction of A20 plants but not plants from the A17 accession^33^. Thus*,* the *S. meliloti* C307 and *M. truncatula* A20 strain-host combination functions as a control while screening through candidate peptidases*.*

**Figure 1:**
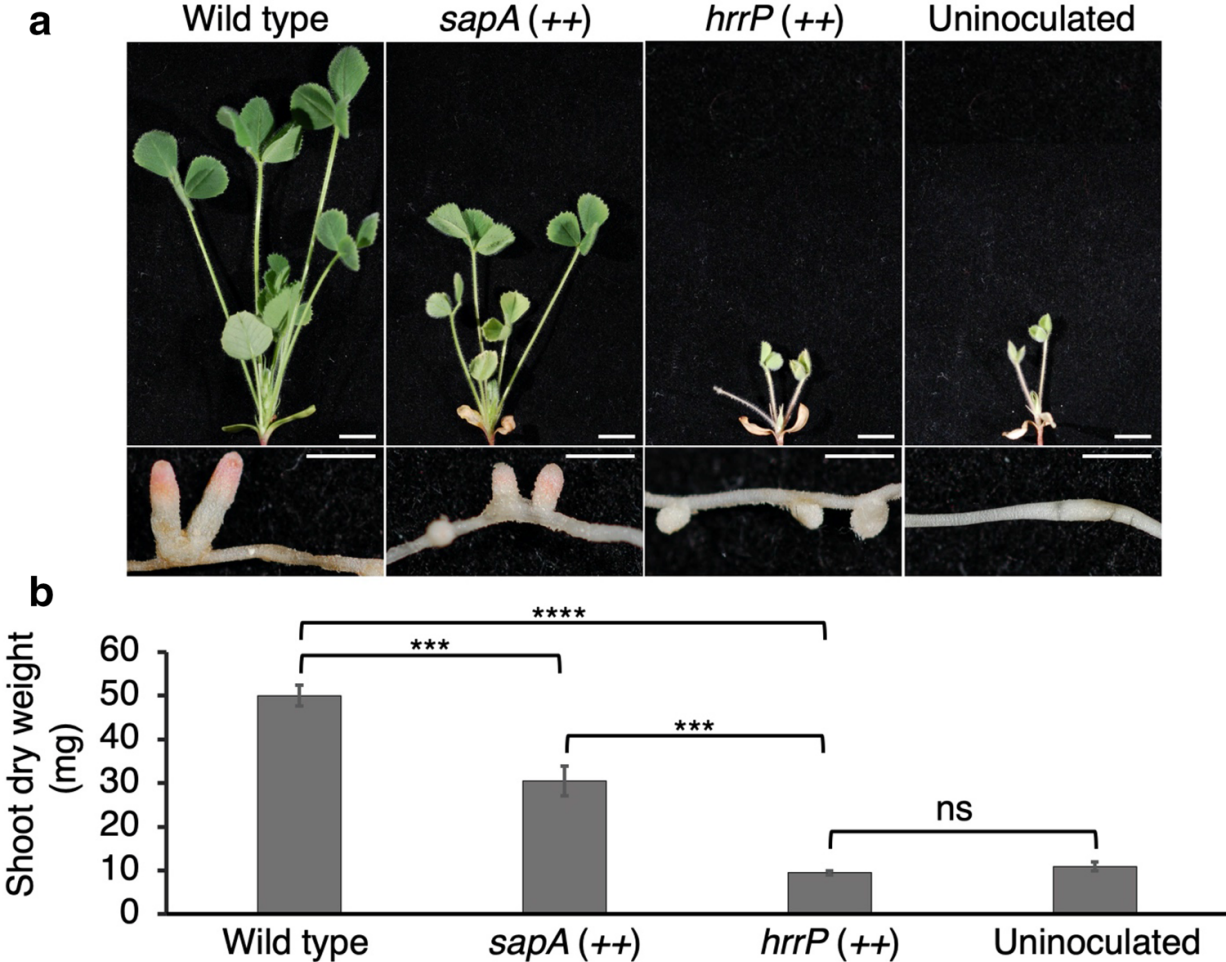
Effects of over-expressing peptidases on plant fitness. (**a**) Representative plants and nodules from each condition were harvested 28 dpi. Scale bars in shoot and nodule pictures are 1 cm and 0.25 cm, respectively. (**b**) Shoot dry mass was determined using the average masses of 12 plants of each condition harvested 28 dpi. For statistical analysis, a one-way ANOVA with a post-hoc Dunnett’s T3 test was performed. Significance levels are indicated (ns = not significant, ***P < 0.001, ****P < 0.0001). Standard error of the mean is represented in each bar.

To test a given peptidase overexpression strain, 12 M*. truncatula* A20 plants were inoculated and grown for 28 days before measuring plant fitness. Empty-vector, *hrrP-*overexpression, and no-inoculum controls were included in each experiment. The 28 candidate peptidases screened in this study (Table 1) represent a variety of proteolytic mechanisms and cellular localizations. Almost all peptidases tested did not have a reproducibly noticeable impact on plant fitness compared to controls; however, overexpression of *SMc00451* (referred to hereafter as *sapA*) led to a considerable decrease in shoot dry weight and nodule size in multiple independent experiments (Fig. 1).

**Table 1:** Putative peptidases screened in *M. truncatula* A20 plants in this study.

Gene	Function	Predicted localization	Fixation phenotype
*SMa0142*	Extracellular serine protease	Cytoplasmic	+
*SMa1126*	Protease M50	Cytoplasmic membrane	+
*SMa1128*	DegP4 protease-like; S1C	Periplasmic	+
*SMa1292*	Peptidase U32	Unknown	+
*SMa1329*	Peptidase; M24	Cytoplasmic	+
*SMb20434*	Probable Xaa-Pro dipeptidase	Cytoplasmic	+
*SMb20697*	Putative peptidase; Peptidase M20	Unknown	+
*SMb21002*	Putative methionine aminopeptidase; Peptidase M24	Cytoplasmic	+
*SMb21495*	Hypothetical protein; degradation of proteins	Unknown	+
*SMc00451* (*sapA*)	Probable processing protease; M16 peptidase	Cytoplasmic	±
*SMc00857*	Probable proteinase; Peptidase S14/S49	Cytoplasmic membrane	+
*SMc01001*	Hypothetical transmembrane protein; Peptidase S54	Cytoplasmic membrane	+
*SMc01135*	Putative protease IV transmembrane protein; Peptidase S49	Cytoplasmic membrane	+
*SMc01438*	Probable serine protease; Peptidase S1C	Periplasmic	+
*SMc01524*	Putative dipeptidase; Peptidase M19	Cytoplasmic	+
*SMc01905*	Probable ATP-dependent LA protease; Peptidase S16	Cytoplasmic	+
*SMc02095*	Zinc metalloprotease M50	Cytoplasmic membrane	+
*SMc02432*	Hypothetical transmembrane protein; Peptidase M23B	Unknown	+
*SMc02547*	Putative proline iminopeptidase; Peptidase S33	Cytoplasmic	+
*SMc02577*	Probable heat shock protein	Cytoplasmic	+
*SMc02825*	Probable aminopeptidase; Peptidase M17	Cytoplasmic	+
*SMc03286*	Serine protease	Unknown	+
*SMc03768*	Hypothetical; Peptidase, trypsin-like serine and cysteine	Unknown	+
*SMc03769*	Serine protease; S1B	Unknown	+
*SMc03783*	Putative c-terminal processing protease; Peptidase S41A	Cytoplasmic membrane	+
*SMc03802*	ATP-dependent protease; Peptidase S16	Cytoplasmic	+
*SMc04091*	Putative protease transmembrane protein; Peptidase M48	Cytoplasmic membrane	+
*SMc04352*	Hypothetical; transglutaminase-like cysteine peptidase	Unknown	+

**Figure 2:**
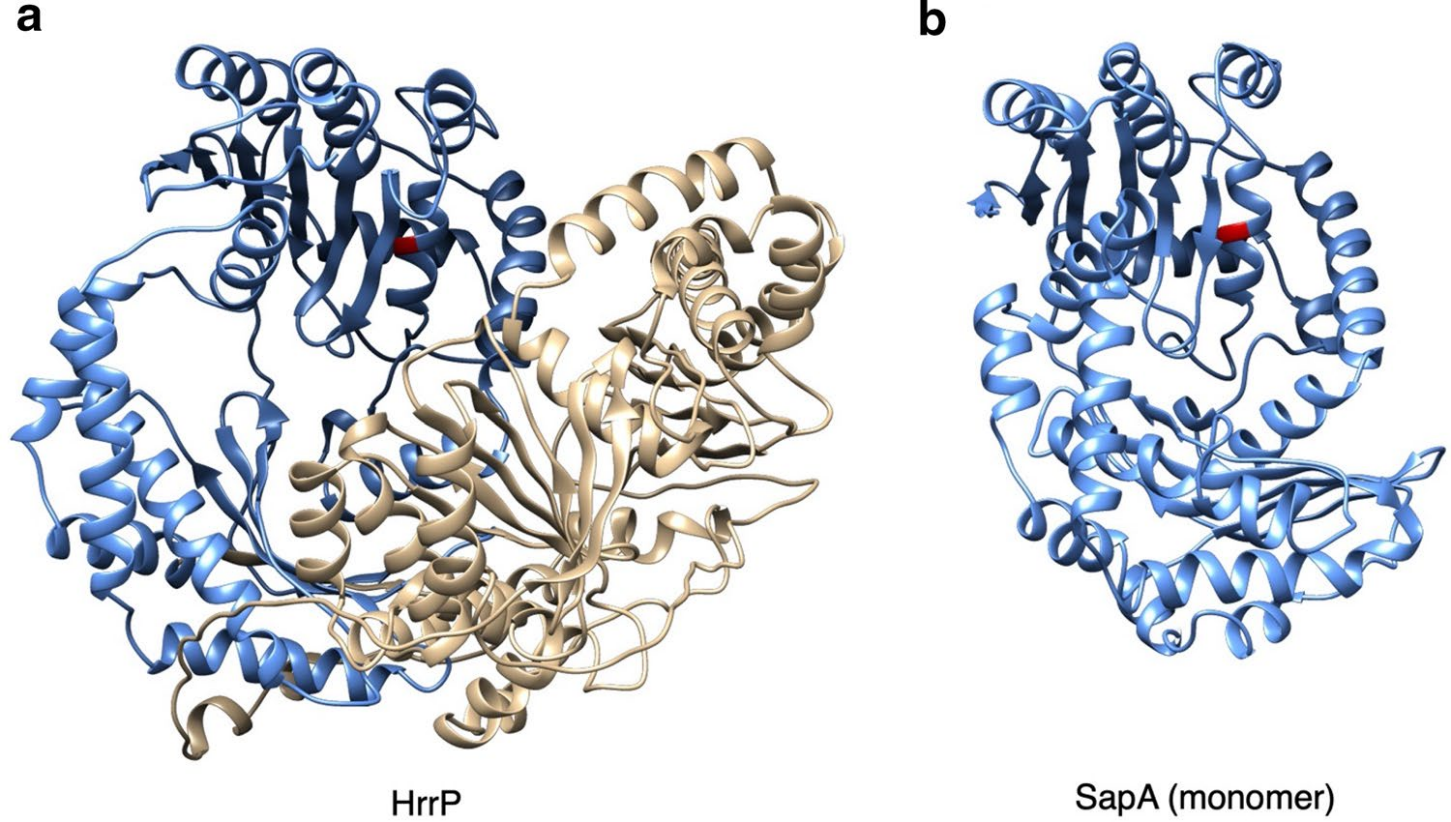
Predicted structures of HrrP and SapA. (**a**) Structures of HrrP and SapA were modelled with I-Tasser and rendered by Chimera (closest PDB structural analogs for each protein were ID codes 6OFS and 1HR6 respectively). The two roughly symmetrical domains in HrrP are colored in blue and gold. (**b**) Though SapA is predicted to function as a homodimer it is depicted above in its monomeric form, colored blue. The active sites in both HrrP and SapA (E62 and E50, respectively), are shown in red.

Homologs of *sapA* are found in all 23 fully sequenced *S. meliloti* genomes in the NCBI database. It encodes an M16 family zinc metallopeptidase and is, based on an NCBI protein BLAST search, the closest homolog encoded in the core *S. meliloti* genome to HrrP. SapA is predicted to form a homodimeric complex that takes on a clamshell structure, characteristic of the M16B subfamily, whereas HrrP is a monomeric fusion of two domains connected by a linker, characteristic of the M16A and M16C subfamilies^37^. SapA has similarity to both halves of HrrP: a pairwise amino acid sequence alignment of SapA with the N-terminal half of HrrP shows 30% identity with 47% coverage, and alignment with the C-terminal half shows 23% identity and 62% coverage. Despite the difference in polypeptide length, their relatedness can be seen in a phylogenetic tree containing four SapA and four HrrP homologs, with a more distantly related sinorhizobial M16 peptidase outgroup (Supplementary Fig. S2). Predicted protein structures of HrrP and SapA underscores their similarity (Fig. 2).

### Degradation of NCR peptides by SapA in vitro

The HrrP peptidase is known to degrade NCR peptides in vitro*,* with degradation occurring much more rapidly under reducing conditions where peptide disulfide linkages are prevented^38^. This is consistent with the predicted localization of HrrP in the bacterial cytoplasm (a reducing environment). In testing the ability of SapA to degrade NCR peptides, we began by addressing the question of whether the peptidase is likely to encounter peptides in the bacterial cytoplasm or extracytoplasmically. Using predictive tools to assess the subcellular localization of SapA, analysis was carried out with SignalP-5.0^39^, DeepSig^40^, and TMHMM 2.0^41^. This analysis indicated a lack of a secretion signal or transmembrane segment in the N-terminal region of SapA. Additionally, in the predicted structural model of SapA (Fig. 2), the N-terminus maps to a structurally conserved 6-stranded ß-sheet, strongly suggesting that it folds into the protein’s globular structure and does not mediate trafficking to an extracytoplasmic location.

**Figure 3:**
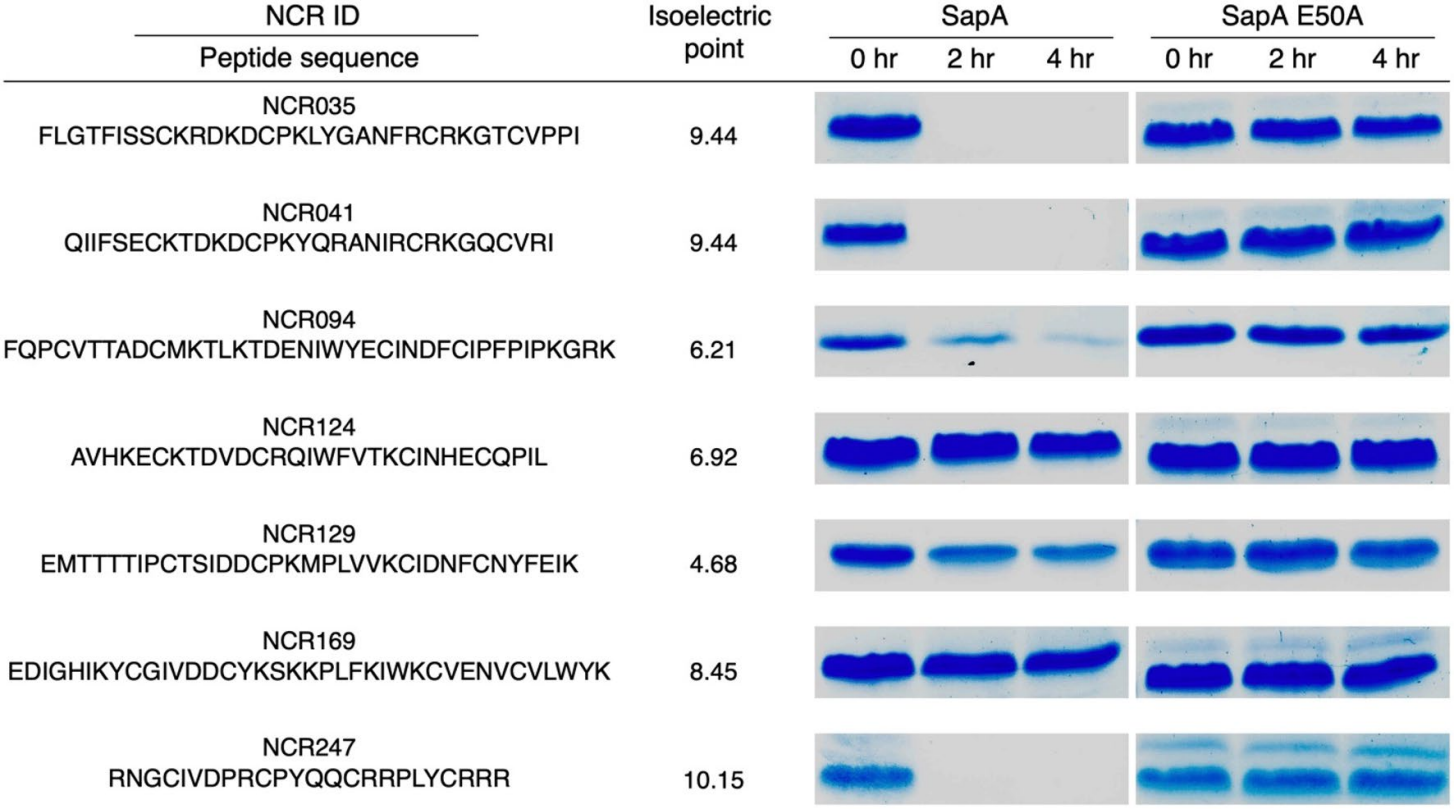
Degradation of NCR peptides by SapA in vitro. SapA and a catalytically inactive version (SapA E50A) were each incubated with several different NCR peptides individually and analyzed via tricine gel at 0, 2, and 4 h. Images of representative experiments are shown above though each peptide was tested for degradation in at least three separate experiments with equivalent results. Full-length gels are presented in Supplementary Fig. S3.

With SapA likely to encounter NCR peptides in the cytoplasm, we purified recombinant SapA and incubated it individually with several NCR peptides under reducing conditions. A portion of each reaction was stopped at 0-, 2-, and 4-h time points to observe degradation over time via gel electrophoresis (Fig. 3). Most peptides were at least partially degraded and 3 were fully degraded within 2 h. A variant form of SapA (E50A), which is presumably catalytically inactive, was purified and tested for activity against NCR peptides in the same manner. This mutant showed no evidence of peptide degradation. The NCR peptides used in this experiment span a range of amino acid lengths and isoelectric points, but all had four similarly spaced cysteine residues. While it is difficult to explain the peptide substrate specificity based on sequence or other chemical properties, it is notable that the same peptides degraded by SapA were also the most readily degraded by HrrP^33^. NCR peptide degradation is likely not a universal property of metallopeptidases, as evidenced by our observation that *S. meliloti* SMb20697, a putative M20 metallopeptidase, was purified and tested in the same conditions with no observable NCR peptide degradation (see Supplementary Fig. S4).

To test whether NCR peptide degradation by SapA is specified by peptide sequence, we incubated SapA with NCR035 (a readily degraded substrate) and three NCR035 variants with scrambled sequences. These sequences were designed to maintain overall amino acid composition. The results at 0- and 2-h time points indicate that SapA exhibits selectivity for NCR035 over the randomized sequence variants (see Supplementary Fig. S5).

### Expression pattern of *sapA*

**Figure 4:**
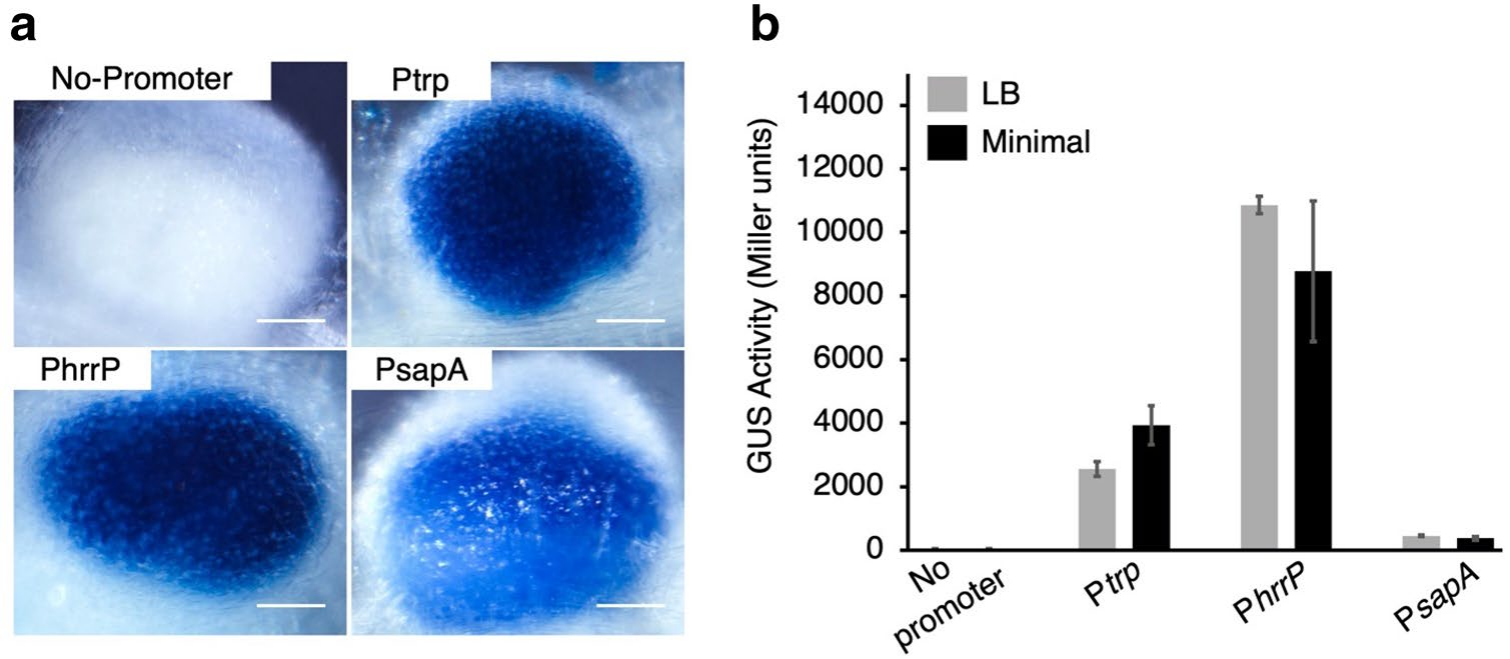
Expression pattern of *sapA *in vivo and in vitro. Promoter-*gus* fusion strains were made to determine if the promoter for *sapA* is active in nodules (**a**) or in free-living cells (**b**). In (**a**), nodules were harvested 7 dpi and stained for GUS activity. Scale bars are 100 μm. In (**b**), Miller assays were performed on three biological replicates of each condition. Error bars correspond to 95% confidence intervals.

To test whether *sapA* is expressed in nodules, we fused the *sapA* promoter (P*sapA*) to a *gus* reporter gene and followed reporter gene activity in nodules and free-living cells. For assessment of nodule expression, *M. truncatula* A20 plants were infected with promoter-*gus* strains, nodules were harvested 7 days post-inoculation (dpi), and these were stained to visualize GUS activity (Fig. 4). P*sapA*, as well as both positive controls (P*trp* and P*hrrP*) displayed GUS activity in nodules, while no GUS activity was observed in the absence of a promoter. Similar expression patterns were seen in nodules harvested and stained at 10 and 14 dpi (not shown). To monitor P*sapA* activity in free-living conditions, we performed Miller assays on three biological replicates of each promoter-*gus* strain in both rich and minimal media. In contrast to nodules, P*sapA* displayed very low activity in either free-living condition, while positive controls (P*trp* and P*hrrP*) were both active in both media. These data indicate that *sapA* expression is relatively low under free-living conditions and at least moderately high in nodules.

## Discussion

Prior to symbiotic nitrogen fixation, *M. truncatula* compels rhizobia to terminally differentiate, in part through the activity of secreted NCR peptides. It was previously shown that the plasmid-encoded peptidase, HrrP, enables endosymbionts to counteract this pressure, by selectively degrading a portion of these peptides^33^. The *hrrP* gene is only found in a small fraction of *Sinorhizobium* isolates^34^. The current study explores the possibility that HrrP involvement in modulating bacteroid differentiation reflects a broader phenomenon involving peptidases present in all or most strains of a rhizobial species. Peptidases encoded in the *S. meliloti* 1021 genome were screened for candidates which, when overexpressed, would negatively impact plant benefits from the symbiosis. Of the 28 peptidases examined, homologs for 23 of them are found in genomes of the 23 fully sequenced strains of *S. meliloti* in the NCBI database. Of these 28 overexpressed peptidases, SapA was the only one that exhibited reproducible symbiotic effects. It is notable that SapA is the closest homolog of HrrP from the core *S. meliloti* genome, suggesting that this M16 metallopeptidase family is particularly suited to the degradation of peptides in the NCR family. Indeed, M16 peptidases are known to accommodate substrate peptides in a size range consistent with NCR peptides^37^.

Based on data from a tissue-specific transcriptomic study, each of the 131 peptidases identified in this study could be detected in nodules, with varying patterns of expression across nodule zones^42^. More than half of these peptidases are expressed at their highest mRNA levels in the interzone (IZ) or zone III regions where bacteroid development and nitrogen fixation occur. Expression of *sapA* also falls in this category, and in agreement with the transcriptomic data, we show that *sapA* expression is readily detectable throughout the nodule. Similarly, the vast majority of NCR peptides, including the NCR peptides degraded by SapA in vitro, are expressed by the host plant at their highest levels where bacteroid development and nitrogen fixation occurs^42^. Two of the NCR peptides degraded by SapA (NCR247 and NCR035) have been shown to significantly affect bacterial cell division, membrane permeability, and DNA replication *in vitro*^24,26,43^. That NCR peptides can have such profound effects on rhizobia, while being susceptible to degradation by conserved rhizobial peptidases, supports a model in which the precise cocktail of peptides and the precise level of peptidase activity are vital determinants of symbiotic compatibility.

It should be considered that NCR peptides are exogenously introduced to developing rhizobia (through a host secretory apparatus), and the rhizobial peptidases that degrade them are likely to be localized in the bacterial cytoplasm. Thus, the peptides must enter bacterial cells in order to be destroyed. This may explain the seemingly counterintuitive observation that the conserved rhizobial peptide uptake transporter, BacA, imports NCR peptides and protects rhizobia from their antimicrobial effects^44,45^. Transporter-mediated uptake likely hastens the delivery of NCR peptides to cytoplasmic bacterial degradation machinery before they can interact with and cause damage to cell membranes. Consistent with this model, BacA expression is known to be upregulated while bacteroid maturation is underway in the nodule^46^. While it is possible for developing bacteroids to secrete peptidases to meet their NCR peptide substrates extracellularly, this strategy may be less effective due to the more structured state of NCR peptides in oxidizing environments. A study on the HrrP peptidase showed that NCR peptides are nearly impervious to degradation when in their disulfide-bonded (oxidized) state^38^. By delivering these peptides to the reducing environment of the cytoplasm, the disulfide-associated protection is likely lost, making this a more ideal location for degradation.

We present evidence, based on peptide motif-finding algorithms and structural modelling, that SapA (like HrrP) is a cytoplasmic enzyme and, as such, can potentially inactivate imported NCR peptides. From our analysis, it is clear that some peptides are more susceptible to degradation than others; though there is no obvious correlation amongst susceptible peptides based on general properties such as isoelectric point, length, or cysteine positioning. Comparing SapA-mediated degradation of NCR035 and several of its scrambled variants, we observe that the native sequence is more actively targeted than sequence variants that have the same overall amino acid composition. This suggests that rhizobial peptidases recognize substrate peptides with some degree of sequence specificity. Analysis of substrate specificity in an M16 peptidase isolated from *Bacillus halodurans* revealed a preference for aromatic residues upstream of the initial cleavage site^37,47^. A possible explanation for the differences in degradation of NCR035 and the scrambled variants, therefore, may be the varied positioning of aromatic residues in these peptides. More focused studies in which residues with specific properties are manipulated could shed more light on these kinds of questions.

The discovery of *sapA* came about through screening a small fraction of the total peptidases in *S. meliloti* strain 1021. Continued efforts to screen for *sap* genes will likely allow for the discovery of an ensemble of rhizobial peptidases that cooperate to modulate symbiotic transactions with the plant host. We likewise suspect that no single peptidase will prove to have a dominant influence on symbiosis based on loss-of-function phenotypes. Thus, continuing the analysis based on overexpression phenotypes will likely be most productive. Considering that *sapA* overexpression yields modest symbiotic defects, one can imagine using this as a sensitized background for carrying out further screening.

## Materials and methods

### Bacterial growth conditions

*E. coli* and *S. meliloti* strains are described in Supplementary Table S2 and were grown in LB (lysogeny broth) at 37 °C and 30 °C respectively and supplemented with the following antibiotics, as appropriate: streptomycin (Sm, 200 or 100 μg/ml), neomycin (Nm, 100 or 50 μg/ml), kanamycin (Km, 30 μg/ml or 15 μg/ml). Strains of *E. coli* used for purifying peptidases were grown at 30 °C. The defined medium used for growth of *S. meliloti* was composed of 0.5% sucrose, 50 mM NaCl, 10 mM KH_2_PO_4_ (pH 7), 10 mM NH_4_Cl, 2 mM MgSO_4_, 215 μM CaCl_2_ • 2H_2_O, 25 μM EDTA, 25 μM FeCl_3_, 12 μM MnSO_4_, 7 μM ZnSO_4_ • 7H_2_O, 3 μM H_3_BO_3_, 1.6 μM nicotinic acid, 1 μM CuSO_4_, 970 nM pyridoxine HCl, 840 nM CoCl_2_, 840 nM pantothenic acid, 826 nM Na_2_MoO_4_, 820 nM biotin, 600 nM thiamine HCl, 530 nM riboflavin, 450 nM folic acid.

### Identification of putative core genome-encoded peptidases

The *S. meliloti* 1021 online genome database includes annotations from Interpro, PubMed, Swiss-Prot, and trEMBL as well as enzyme codes and functional classifications for most genes. Enzyme codes, and generic terms including “peptidase”, “protease”, “hydrolase” and others, as well as variations on those terms, were used to compile a large list of candidate peptidases. This list was refined and compared with all peptidases for *S. meliloti* 1021 found in the *MEROPS* database to obtain a final set of 131 peptidases. Any enzyme annotated as “acting on C-N (but not peptide) bonds" or classified with an Enzyme Commission number other than 3.4 were removed. Conserved hypothetical proteins with a generic hydrolase or hydrolase-like annotation were included in the set.

### Screen for symbiotically relevant peptidases

Genes for candidate peptidases were cloned into the overexpression vector pPG013 (plasmids are listed in Supplementary Table S3) using primers listed in Supplementary Table S4. Clones were verified by Sanger sequencing and mated into *S. meliloti* C307 before inoculating plants. The pPG013 plasmid was constructed by assembling four fragments: the first fragment contained, pVS1 *repA*, *staA*, and *oriV*, elements; the second fragment contained, *kanR* and the p15A *oriV*; the third fragment contained, the RK2 mobilization element, *oriT*; finally, a multiple cloning site and the *hrrP* promoter were added. A map for this plasmid is provided in Supplementary Fig. S1 with sequence details at the end of the Supplementary Information file.

### Plant growth conditions and determining shoot dry weight

The A20 seeds used in these experiments were kindly provided by the Sharon Long laboratory, Stanford University, Stanford, California. For each experimental condition, 12 seeds were planted in a sterile mixture of washed Turface and vermiculite (4:1 ratio) and watered with a defined nutrient medium lacking nitrogen. This medium is composed of 1 mM KH_2_PO_4_ (pH 7), 2 mM MgSO_4_, 2 mM CaCl_2_ • 2H_2_O, 50 μM Na_2_ EDTA, 50 μM FeSO_4_, 32 μM HBO_3_, 3 μM MnSO_4_, 626 nM CuSO_4_, 414 nM Na_2_MoO_4_, 348 nM ZnSO_4_ • 7H_2_O, 84 nM CoCl_2_. Plants were inoculated after 2 days and allowed to grow 28 days after inoculation before harvesting and imaging. Representative plants were imaged using a Nikon D50 SLR camera. Harvested shoots were placed into individual coin envelopes and incubated at 62 °C for 4 days before weighing.

### Phylogenetic comparison of SapA and HrrP homologs

Phylogenetic analysis was carried out using the Phylogeny.fr platform^48,49^ and comprised the following steps: Sequences were aligned with ClustalW (v2.1)^50^. After alignment, ambiguous regions were removed with Gblocks (v0.91b)^51^ using the following parameters: minimum length of a block after gap cleaning was 5, positions with a gap in less than 50% of the sequences were selected in the final alignment if they were within an appropriate block, all segments with contiguous nonconserved positions larger than 8 were rejected, and the minimum number of sequences for a flank position was 55%. The phylogenetic tree was reconstructed using the Bayesian inference method implemented in the MrBayes program (v3.2.6)^52^. The number of substitution types was fixed to 6. The Poisson model was used for amino acid substitution, while rate variation across sites was fixed to "invgamma”. Four Markov Chain Monte Carlo (MCMC) chains were run for 100,000 generations, sampling every 10 generations, with the first 5000 sampled trees discarded as "burn-in". Finally, a 50% majority rule consensus tree was constructed. Graphical representation of the phylogenetic tree was rendered using TreeDyn (v198.3)^53^. The GenBank accession numbers for the analyzed proteins are: CAC45492.2, ABR59391.1, ACP24338.1, AAK86595.2, PDT36621.1, AJT61688.1, AEG58046.1, ACI55061.1, and ACE91149.1

### Protein structure modelling and purification

Predicted structural models of SapA and HrrP were generated with iTASSER^54^ and rendered using UCSF Chimera^55–57^. Various inserts containing a 6-His tag adjacent to a protein coding sequence were cloned into the protein expression vector pJG729 (protein expression plasmids are listed in Supplementary Table S3) using primers listed in Supplementary Table S4. This plasmid is a slightly modified version of pSX2 (Scarab Genomics) that includes a ColE1 *oriV*, *kanR*, *oriT*, *lacI* repressor, and a T5/*lac* promoter upstream of the multiple cloning site. A map for the empty pJG729 plasmid is provided in Supplementary Fig. S1 with relevant insert sequences listed at the end of the Supplementary Information file. NiCO21 (DE3) *E. coli* cells harboring a protein expression plasmid were grown overnight at 37 °C. Saturated culture was transferred to an LB-Km (15 μg/ml) flask and grown at 30 °C for 1 h. Cultures were then induced with 300 μM isopropyl β-d-1-thiogalactopyranoside (IPTG) and grown at 30 °C for an additional 6 h before centrifuging at 8,000 RPM for 15 min at 4 °C in a Sorvall RC5C Plus (GSA rotor) or Thermo Scientific Sorvall Lynx 4000 (F12-6 × 500 rotor), removing supernatant, and freezing at − 80 °C. Pellets were resuspended in lysis buffer (1 mM EDTA pH 8, 20 mM imidazole, 0.2% Triton X-100, 300 mM NaCl, 50 mM HEPES pH 7.8, and 0.5 mg/ml lysozyme) and incubated on ice for ~ 1 h before sonication. Disrupted cells were centrifuged at 14,000 RPM for ~ 45 min at 4 °C in a benchtop microfuge and supernatant was transferred to a tube containing Ni–NTA agarose beads (Qiagen) and rotated for 1 h. Beads were washed 3 times with wash buffer (35 mM imidazole, 300 mM NaCl, 50 mM HEPES pH 7.8) and pelleted. Elution buffer (270 mM Imidazole, 100 mM NaCl, and 50 mM HEPES pH 7.8) was then added to the beads and allowed to incubate for up to 1 h before pelleting. Supernatant containing the enzyme of interest was transferred to a new tube with 50 μl chitin resin (NEB) and rotated 50 min for removal of contaminant proteins. Beads were pelleted and the enzyme-containing supernatant was buffer exchanged in 50 mM KH_2_PO_4_ pH 7.2, using Bio-Gel P-6 DG media (BioRad) then stored in 20% glycerol at − 80 °C. Concentration of protein was measured using a Bradford assay with known protein standards.

### In vitro NCR peptide degradation assays

Peptides were synthesized commercially from GenScript and prepared at a 1 mg/ml concentration. Approximately 4 μg of purified SapA (or the E50A variant) was incubated with 4 μg of peptide in buffer composed of 10 mM DTT, 50 mM KH_2_PO_4_ pH 7.2, and 1 μM ZnSO_4_ • 7H_2_O. At 0-, 2-, and 4-h time points, a portion of the reaction was stopped by adding sample buffer and transferring to ice. Sample buffer contained 32% glycerol, 150 mM Tris pH 6.8, 20 mM EDTA, 40 mg/ml SDS, 15.5 mg/ml DTT, and 0.3% bromophenol blue. Following the addition of stop buffer, samples were moved to an 85 °C heat block for 3 min and then placed back on ice before visualization on a tricine gel. In the experiment where SapA and NCR035 (or scrambled variants of NCR035) were tested, less enzyme was used (approximately 2 μg of purified SapA and 4 μg of peptide), to make subtle differences in degradation easier to visualize.

### Quantification of GUS activity

Promoter-*gus* fusion strains were made by cloning various inserts into the *gus* expression vector pPG178 (promoter-*gus* plasmids are listed in Supplementary Table S3) using primers listed in Supplementary Table S4. This plasmid has the same backbone as pPG013 but, unlike pPG013, various promoters were cloned upstream of the *gus* reporter gene. A map of the empty plasmid is available in Supplementary Fig. S1 followed by relevant insert sequences. Cultures used to measure GUS activity were grown in three biological replicates. To each reaction, 20 μl of cells in log phase (OD_600_ 0.6–1.1) were added to a reaction composed of 520 μl basal buffer (60 mM Na_2_HPO_4_, 40 mM NaH_2_PO_4_ pH 7), 3 μl ß-mercaptoethanol, 3 μl 20% tween, 20 μl CHCl_3_, and 60 μl *p*-Nitrophenyl α-D-glucopyranoside (8 mg/ml). Reactions proceeded 15 min before stopping with 600 μl 1 M Na_2_CO_3_ and taking OD_405_ measurements.

### Nodule staining

Harvested nodules (7 dpi) were fixed in 90% acetone for 1 h at − 20 °C. Following fixation, nodules were washed twice in 100 mM Na_2_HPO_4_ pH 7.2 then immersed in fixative (100 mM Na_2_PO_4_ pH 7.2, 0.5 mg/mL 5-bromo-4-chloro-3-indolyl-beta-D-glucuronic acid (X-Gluc), 0.5 mM K_3_Fe(CN)_6_, 0.5 mM K_4_Fe(CN)_6_) for 1 h 45 min at 37 °C. Nodules were then washed for 5 s twice in separate containers of 100 mM Na_2_HPO_4_ pH 7.2, bleached for 5 min, and again washed for 5 s twice in separate containers of 100 mM Na_2_HPO_4_ pH 7.2 before storing in ddH_2_O. Stained nodules were visualized with an Olympus SZX stereomicroscope with an SZX-TBI tilting binocular tube, WHS10X-H/22 eyepiece, and DFPLAPO 1X PF objective. Images were taken using an Olympus U-TV1X-2/U-CAMD3SZX12 microscope camera.

## Supplementary Information

Supplementary Information 1.

## Data Availability

The use of plant material in the present study compiles with international, national, and institutional guidelines and, as a model organism, can be obtained from various laboratories and seed companies worldwide.

## References

[1] Peters N, Frost J, Long S (1986). A plant flavone, luteolin, induces expression of rhizobium meliloti nodulation genes. Science.

[2] Subramanian S, Stacey G, Yu O (2007). Distinct, crucial roles of flavonoids during legume nodulation. Trends Plant Sci..

[3] Hartwig UA, Phillips DA (1991). Release and modification of nod-gene-inducing flavonoids from alfalfa seeds. Plant Physiol..

[4] Roche P (1996). The common nodabc genes of *rhizobium meliloti* are host-range determinants. Proc. Natl. Acad. Sci. USA.

[5] Wais RJ, Keating DH, Long SR (2002). Structure-function analysis of nod factor-induced root hair calcium spiking in rhizobium-legume symbiosis. Plant Physiol..

[6] Fisher RF, Long SR (1992). Rhizobium–plant signal exchange. Nature.

[7] Leigh JA, Signer ER, Walker GC (1985). Exopolysaccharide-deficient mutants of *rhizobium meliloti* that form ineffective nodules. Proc. Natl. Acad. Sci. USA.

[8] Perret X, Staehelin C, Broughton WJ (2000). Molecular basis of symbiotic promiscuity. Microbiol. Mol. Biol. Rev..

[9] Bauer WD (1981). Infection of legumes by rhizobia. Annu. Rev. Plant Physiol..

[10] Hassan S, Mathesius U (2012). The role of flavonoids in root–rhizosphere signalling: Opportunities and challenges for improving plant–microbe interactions. J. Exp. Bot..

[11] Mendis HC, Madzima TF, Queiroux C, Jones KM (2016). Function of succinoglycan polysaccharide in *sinorhizobium meliloti* host plant invasion depends on succinylation, not molecular weight. MBio.

[12] Jones KM (2012). Increased production of the exopolysaccharide succinoglycan enhances *sinorhizobium meliloti* 1021 symbiosis with the host plant *medicago truncatula*. J. Bacteriol..

[13] Fournier J (2008). Mechanism of infection thread elongation in root hairs of medicago truncatula and dynamic interplay with associated rhizobial colonization. Plant Physiol..

[14] Gage DJ (2004). Infection and invasion of roots by symbiotic, nitrogen-fixing rhizobia during nodulation of temperate legumes. Microbiol. Mol. Biol. Rev..

[15] Liu J, Yang S, Zheng Q, Zhu H (2014). Identification of a dominant gene in medicago truncatula that restricts nodulation by *sinorhizobium meliloti* strain rm41. BMC Plant Biol..

[16] Crook MB (2012). Rhizobial plasmids that cause impaired symbiotic nitrogen fixation and enhanced host invasion. Mol. Plant Microbe Interact..

[17] Wang Q (2017). Host-secreted antimicrobial peptide enforces symbiotic selectivity in *medicago truncatula*. Proc. Natl. Acad. Sci. USA.

[18] Yang S (2017). Microsymbiont discrimination mediated by a host-secreted peptide in medicago truncatula. Proc. Natl. Acad. Sci. USA.

[19] Starker CG, Parra-Colmenares AL, Smith L, Mitra RM, Long SR (2006). Nitrogen fixation mutants of *medicago truncatula* fail to support plant and bacterial symbiotic gene expression. Plant Physiol..

[20] Bourcy M (2013). Medicago truncatula dnf2 is a pi-plc-xd-containing protein required for bacteroid persistence and prevention of nodule early senescence and defense-like reactions. New Phytol..

[21] Mergaert P (2003). A novel family in *medicago truncatula* consisting of more than 300 nodule-specific genes coding for small, secreted polypeptides with conserved cysteine motifs. Plant Physiol..

[22] Maróti G, Downie JA, Kondorosi É (2015). Plant cysteine-rich peptides that inhibit pathogen growth and control rhizobial differentiation in legume nodules. Curr. Opin. Plant Biol..

[23] Arnold MFF (2017). Genome-wide sensitivity analysis of the microsymbiont *sinorhizobium meliloti* to symbiotically important, defensin-like host peptides. MBio.

[24] Farkas A (2014). *Medicago truncatula* symbiotic peptide ncr247 contributes to bacteroid differentiation through multiple mechanisms. Proc. Natl. Acad. Sci. USA.

[25] Penterman J (2014). Host plant peptides elicit a transcriptional response to control the *sinorhizobium meliloti* cell cycle during symbiosis. Proc. Natl. Acad. Sci. USA.

[26] Van de Velde W (2010). Plant peptides govern terminal differentiation of bacteria in symbiosis. Science.

[27] Mikuláss KR (2016). Antimicrobial nodule-specific cysteine-rich peptides disturb the integrity of bacterial outer and inner membranes and cause loss of membrane potential. Ann. Clin. Microbiol. Antimicrob..

[28] Montiel J (2017). Morphotype of bacteroids in different legumes correlates with the number and type of symbiotic ncr peptides. Proc. Natl. Acad. Sci. USA.

[29] Mergaert P (2006). Eukaryotic control on bacterial cell cycle and differentiation in the rhizobium–legume symbiosis. Proc. Natl. Acad. Sci. USA.

[30] Wang D (2010). A nodule-specific protein secretory pathway required for nitrogen-fixing symbiosis. Science.

[31] Kim M (2015). An antimicrobial peptide essential for bacterial survival in the nitrogen-fixing symbiosis. Proc. Natl. Acad. Sci. USA.

[32] Horváth B (2015). Loss of the nodule-specific cysteine rich peptide, ncr169, abolishes symbiotic nitrogen fixation in the *medicago truncatula* dnf7 mutant. Proc. Natl. Acad. Sci. USA.

[33] Price PA (2015). Rhizobial peptidase hrrp cleaves host-encoded signaling peptides and mediates symbiotic compatibility. Proc. Natl. Acad. Sci. USA.

[34] Wendlandt CE (2021). Decreased coevolutionary potential and increased symbiont fecundity during the biological invasion of a legume-rhizobium mutualism. Evolution.

[35] Arkin AP (2018). Kbase: The united states department of energy systems biology knowledgebase. Nat. Biotechnol..

[36] Yu NY (2010). Psortb 3.0: Improved protein subcellular localization prediction with refined localization subcategories and predictive capabilities for all prokaryotes. Bioinformatics.

[37] Aleshin AE (2009). Crystal and solution structures of a prokaryotic m16b peptidase: An open and shut case. Structure.

[38] Shabab M (2016). Disulfide cross-linking influences symbiotic activities of nodule peptide ncr247. Proc. Natl. Acad. Sci. USA.

[39] Almagro Armenteros JJ (2019). Signalp 5.0 improves signal peptide predictions using deep neural networks. Nat. Biotechnol..

[40] Savojardo C, Martelli PL, Fariselli P, Casadio R (2017). Deepsig: Deep learning improves signal peptide detection in proteins. Bioinformatics.

[41] Krogh A, Larsson B, von Heijne G, Sonnhammer ELL (2001). Predicting transmembrane protein topology with a hidden markov model: Application to complete genomes. J. Mol. Biol..

[42] Roux B (2014). An integrated analysis of plant and bacterial gene expression in symbiotic root nodules using laser-capture microdissection coupled to rna sequencing. Plant J..

[43] Lima RM, Kylarová S, Mergaert P, Kondorosi É (2020). Unexplored arsenals of legume peptides with potential for their applications in medicine and agriculture. Front. Microbiol..

[44] Haag AF (2011). Protection of sinorhizobium against host cysteine-rich antimicrobial peptides is critical for symbiosis. PLoS Biol..

[45] Guefrachi I (2015). Bradyrhizobium bcla is a peptide transporter required for bacterial differentiation in symbiosis with aeschynomene legumes. Mol. Plant Microbe Interact..

[46] Oke V, Long SR (1999). Bacterial genes induced within the nodule during the rhizobium–legume symbiosis. Mol. Microbiol..

[47] Dabonné S (2005). Cloning, expression and characterization of a 46.5-kda metallopeptidase from bacillus halodurans h4 sharing properties with the pitrilysin family. Biochim. Biophys. Acta (BBA) Gen. Subj..

[48] Dereeper A, Audic S, Claverie J-M, Blanc G (2010). Blast-explorer helps you building datasets for phylogenetic analysis. BMC Evol. Biol..

[49] Dereeper A (2008). Phylogeny.Fr: Robust phylogenetic analysis for the non-specialist. Nucleic Acids Res..

[50] Thompson JD, Higgins DG, Gibson TJ (1994). Clustal w: Improving the sensitivity of progressive multiple sequence alignment through sequence weighting, position-specific gap penalties and weight matrix choice. Nucleic Acids Res..

[51] Castresana J (2000). Selection of conserved blocks from multiple alignments for their use in phylogenetic analysis. Mol. Biol. Evol..

[52] Huelsenbeck JP, Ronquist F (2001). Mrbayes: Bayesian inference of phylogenetic trees. Bioinformatics.

[53] Chevenet F, Brun C, Bañuls A-L, Jacq B, Christen R (2006). Treedyn: Towards dynamic graphics and annotations for analyses of trees. BMC Bioinform..

[54] Pettersen EF (2004). Ucsf chimera—A visualization system for exploratory research and analysis. J. Comput. Chem..

[55] Yang J (2015). The i-tasser suite: Protein structure and function prediction. Nat. Methods.

[56] Roy A, Kucukural A, Zhang Y (2010). I-tasser: A unified platform for automated protein structure and function prediction. Nat. Protoc..

[57] Zhang Y (2008). I-tasser server for protein 3d structure prediction. BMC Bioinform..

