# Supplementary material for: A conserved rhizobial peptidase that interacts with host-derived symbiotic peptides
Source: Sci Rep. 2021 Jun 3;11:11779. doi: 10.1038/s41598-021-91394-x (PMC8175422; doi:10.1038/s41598-021-91394-x)
Supplement: Supplementary file 1 — Supplementary Information 1. [file 41598_2021_91394_MOESM1_ESM.docx]

**Supplementary information**

**A conserved rhizobial peptidase that interacts with host-derived symbiotic peptides**

†Alex B. Benedict^1^, †Prithwi Ghosh^1^, Samuel M. Scott^1^, *Joel S. Griffitts^1^

^1^Department of Micro and Molecular Biology, Brigham Young University, Provo, Utah, 84602, USA

†Authors contributed equally to this work

*Corresponding author:

Email: joelg@byu.edu

Phone: 801-422-7997

**Table S1.** List of 131 putative peptidase candidates identified in *S. meliloti* 1021.

| **Gene** | **Function** | **Predicted localization** | **Fixation phenotype** |
| --- | --- | --- | --- |
| SMa0034 | Serralysin-like metalloprotease | Extracellular^a^ | +^b^ |
| SMa0095 | D-aminopeptidase; peptidase S58 | Cytoplasmic | + |
| *SMa0142^c^* | *Extracellular serine protease* | *Cytoplasmic* | + |
| *SMa0275* | *Conserved hypothetical; peptidase C56* | *Cytoplasmic* | Not tested |
| SMa0429 | Conserved hypothetical; peptidase S8/S53 | Cytoplasmic | Not tested |
| *SMa0599* | *Conserved hypothetical; hydrolase-like* | *Unknown* | Not tested |
| SMa0708 | Enolase; M peptidase | Cytoplasmic | Not tested |
| SMa0792 | Putative hydrolase | Unknown | Not tested |
| SMa1126 | Protease M50 | Cytoplasmic membrane | + |
| SMa1128 | DegP4 protease-like; S1C | Periplasmic | + |
| SMa1291 | Protease U32 | Cytoplasmic | Not tested |
| SMa1292 | Peptidase U32 | Unknown | + |
| SMa1327 | Conserved hypothetical; hydrolase activity | Cytoplasmic | Not tested |
| SMa1329 | Peptidase; M24 | Cytoplasmic | + |
| SMa1473 | Proline dipeptidase; M24 | Cytoplasmic | Not tested |
| *SMa1578* | *CpaA2 prepilin peptidase A24A* | *Cytoplasmic membrane* | Not tested |
| SMa1727 | Alpha/beta hydrolase fold protein | Unknown | Not tested |
| SMa1776 | Conserved hypothetical protein; hydrolase-like | Unknown | Not tested |
| SMa1903 | Serine protease | Cytoplasmic | + |
| SMa1968 | Amidotransferase; PFPI-like cystein-proteinase | Cytoplasmic | Not tested |
| SMa1969 | Amidotransferase; PFPI-like cystein-proteinase | Unknown | Not tested |
| SMb20010 | Hypothetical protein; Alpha/beta hydrolase fold | Unknown | Not tested |
| SMb20028 | Hypothetical protein; Peptidase C45 | Unknown | Not tested |
| SMb20212 | Hypothetical protein; PFPI-like cystein-proteinase | Cytoplasmic | Not tested |
| SMb20335 | Conserved hypothetical protein; hydrolase-like | Unknown | Not tested |
| SMb20384 | Membrane dipeptidase; Peptidase M19 | Cytoplasmic | Not tested |
| SMb20434 | Probable Xaa-Pro dipeptidase | Cytoplasmic | + |
| SMb20466 | Putative peptidase; Peptidase M42 | Cytoplasmic | + |
| SMb20547 | Hypothetical protein; Peptidase aspartic | Unknown | Not tested |
| SMb20579 | Beta-ketoadipate enol-lactone hydrolase; Peptidase M14 | Cytoplasmic | Not tested |
| SMb20697 | Putative peptidase; Peptidase M20 | Unknown | + |
| SMb20866 | Conserved hypothetical; Peptidase S8 and S53 | Cytoplasmic | Not tested |
| SMb20925 | Conserved hypothetical membrane protein; Peptidase M50 | Cytoplasmic membrane | Not tested |
| SMb20928 | Putative exported carboxyl-terminal protease; Peptidase S41A | Cytoplasmic | Not tested |
| SMb21002 | Putative methionine aminopeptidase; Peptidase M24 | Cytoplasmic | Not tested |
| SMb21042 | Tripeptide aminopeptidase; Peptidase M20B | Cytoplasmic | Not tested |
| SMb21279 | Putative amidohydrolase; Peptidase M20 | Cytoplasmic | Not tested |
| SMb21424 | Putative acyl esterase; Peptidase S15 | Unknown | Not tested |
| SMb21495 | Hypothetical protein; degradation of proteins | Unknown | + |
| SMb21496 | Putative protease; Serralysin-like metalloprotease | Extracellular | + |
| SMb21538 | Probable phosphonoacetate hydrolase; Peptidase M14 | Cytoplasmic | Not tested |
| SMb21543 | Conserved hypothetical; metallopeptidase | Extracellular | Not tested |
| *SMc00038* | *Diguanylate cyclase/phosphodiesterase; Peptidase* | *Cytoplasmic membrane* | Not tested |
| *SMc00068* | *Probable D-alanyl-D-alanine carboxypeptidase* | *Cytoplasmic membrane* | Not tested |
| *SMc00114* | *Probable protease II oligopeptidase B; Peptidase S9A* | *Unknown* | Not tested |
| SMc00291 | Conserved hypothetical; Hedgehog/DD-peptidase | Unknown | + |
| *SMc00298* | *Putative methionine aminopeptidase; Peptidase M24* | *Cytoplasmic* | Not tested |
| *SMc00360* | *Hypothetical; Predicted transglutaminase-like cysteine peptidase* | *Unknown* | Not tested |
| *SMc00361* | *Hypothetical; Putative AB hydrolase superfamily* | *Cytoplasmic* | Not tested |
| SMc00382 | Probable aminopeptidase; Peptidase M29 | Unknown | Not tested |
| *SMc00451* | *(SapA); Probable processing protease; M16 peptidase* | *Cytoplasmic* | +/- |
| *SMc00512* | *Conserved hypothetical; Peptidase S58* | *Unknown* | Not tested |
| *SMc00539* | *Hypothetical transmembrane protein; Peptidase M23B* | *Unknown* | + |
| *SMc00585* | *Probable aminopeptidase; Peptidase M17* | *Cytoplasmic* | Not tested |
| *SMc00682* | *Putative hippurate hydrolase; M20 peptidase* | *Cytoplasmic* | Not tested |
| SMc00683 | Putative pbp; Serine-type D-Ala-D-Ala carboxypeptidase activity | Cytoplasmic membrane | Not tested |
| SMc00730 | Hypothetical transmembrane protein; Peptidase S54 | Cytoplasmic membrane | Not tested |
| SMc00783 | Hypothetical transmembrane protein; Peptidase M48 | Unknown | Not tested |
| *SMc00814* | *Hypothetical signal peptide protein; Peptidase aspartic* | *Unknown* | Not tested |
| SMc00857 | Probable proteinase; Peptidase S14/S49 | Cytoplasmic membrane | + |
| *SMc00897* | *Hypothetical PmbA protein; Peptidase U62* | *Cytoplasmic* | Not tested |
| SMc00955 | Conserved hypothetical; hydrolase-like | Unknown | Not tested |
| *SMc00960* | *Peptidase U62 family protein* | *Cytoplasmic* | Not tested |
| SMc00996 | Penicillin-binding protein; D-alanyl-D-alanine carboxypeptidase | Cytoplasmic membrane | Not tested |
| *SMc00998* | *Hypothetical signal peptide protein; transglutaminase-like cysteine peptidase* | *Unknown* | Not tested |
| *SMc01001* | *Hypothetical transmembrane protein; Peptidase S54* | *Cytoplasmic membrane* | + |
| *SMc01118* | *Putative peptidase; Peptidase M22* | *Cytoplasmic* | Not tested |
| *SMc01129* | *Probable transmembrane lipoprotein signal peptidase; Peptidase A8* | *Cytoplasmic membrane* | Not tested |
| *SMc01135* | *Putative protease IV transmembrane protein; Peptidase S49* | *Cytoplasmic membrane* | + |
| *SMc01188* | *Penicillin-binding precursor transmembrane protein; serine-type carboxypeptidase* | *Cytoplasmic membrane* | Not tested |
| *SMc01213* | *Hypothetical transmembrane protein; Putative zinc metallopeptidase* | *Cytoplasmic* | Not tested |
| *SMc01280* | *Serine protease S1C* | *Periplasmic* | + |
| *SMc01438* | *Probable serine protease; Peptidase S1C* | *Periplasmic* | + |
| *SMc01440* | *Putative hydrolase serine protease transmembrane protein* | *Cytoplasmic* | Not tested |
| *SMc01441* | *Putative membrane bound protease; hydrolase serine protease* | *Cytoplasmic* | Not tested |
| *SMc01524* | *Putative dipeptidase; Peptidase M19* | *Cytoplasmic* | + |
| *SMc01648* | *Putative hydrolase; Peptidase S15* | *Unknown* | + |
| *SMc01885* | *Putative aminopeptidase P; Peptidase M24* | *Cytoplasmic* | + |
| *SMc01903* | *Probable ATP-dependent CLP protease proteolytic subunit; Peptidase S14* | *Cytoplasmic* | Not tested |
| *SMc01904* | *Probable ATP-dependent CLP protease ATP-binding subunit* | *Cytoplasmic* | Not tested |
| *SMc01905* | *Probable ATP-dependent LA protease; Peptidase S16* | *Cytoplasmic* | + |
| SMc02024 | Conserved hypothetical; Peptidase M20 | Unknown | + |
| *SMc02060* | *Lipoprotein precursor; Peptidase M23B* | *Outer membrane* | Not tested |
| *SMc02095* | *Zinc metalloprotease M50* | *Cytoplasmic membrane* | + |
| *SMc02109* | *Probable ATP-dependent CLP protease ATP-binding subunit; Peptidase S16* | *Cytoplasmic* | Not tested |
| *SMc02110* | *Putative ATP-dependent Clp protease adapter protein clpS1* | *Cytoplasmic* | Not tested |
| *SMc02223* | *Transcriptional regulator; Peptidase S24/S26A/S26B/S26C* | *Cytoplasmic* | Not tested |
| *SMc02256* | *Putative hippurate hydrolase; Peptidase M20* | *Cytoplasmic* | Not tested |
| *SMc02365* | *Probable serine protease; Peptidase S1C* | *Periplasmic* | Not tested |
| *SMc02370* | *Probable aminopeptidase N; Peptidase M1* | *Cytoplasmic* | Not tested |
| SMc02432 | Hypothetical transmembrane protein; Peptidase M23B | Unknown | + |
| SMc02433 | Probable ATP-dependent protease (heat shock protein) | Cytoplasmic | Not tested |
| *SMc02547* | *Putative proline iminopeptidase; Peptidase S33* | *Cytoplasmic* | + |
| *SMc02575* | *Probable heat shock protein* | *Cytoplasmic* | Not tested |
| *SMc02577* | *Probable heat shock protein* | *Cytoplasmic* | + |
| *SMc02653* | *Probable signal peptidase I transmembrane protein; Peptidase S26A* | *Cytoplasmic membrane* | Not tested |
| *SMc02684* | *Hydrolase/peptidase; Peptidase M24* | *Cytoplasmic* | Not tested |
| *SMc02694* | *Putative ATP-dependent Clp protease adapter protein clpS2* | *Cytoplasmic* | Not tested |
| *SMc02720* | *CLP protease proteolytic subunit; Peptidase S14* | *Cytoplasmic* | Not tested |
| *SMc02768* | *Conserved hypothetical protein; Peptidase M* | *Cytoplasmic* | Not tested |
| *SMc02818* | *Conserved hypothetical protein; Alpha/beta hydrolase fold-1* | *Cytoplasmic* | Not tested |
| *SMc02825* | *Probable aminopeptidase; Peptidase M17* | *Cytoplasmic* | + |
| *SMc02827* | *Putative gamma-D-glutamyl-L-diamino acid endopeptidase II, NlpC/P60 family* | *Cytoplasmic* | Not tested |
| *SMc02833* | *Putative murein endopeptidase transmembrane protein; Peptidase M74* | *Periplasmic* | Not tested |
| *SMc02852* | *Putative peptidase; Peptidase M20* | *Cytoplasmic* | Not tested |
| *SMc03230* | *Probable O-sialoglycoprotein endopeptidase; Peptidase M22* | *Extracellular* | Not tested |
| SMc03264 | Putative dipeptidase; Peptidase M19 | Cytoplasmic | Not tested |
| SMc03267 | Putative dipeptidase; Peptidase M19 | Cytoplasmic | Not tested |
| SMc03286 | Serine protease | Unknown | + |
| SMc03768 | Hypothetical; Peptidase, trypsin-like serine and cysteine | Unknown | + |
| SMc03769 | Serine protease; S1B | Unknown | + |
| *SMc03782* | *Hypothetical signal peptide protein; Peptidase M23B* | *Unknown* | Not tested |
| *SMc03783* | *Putative c-terminal processing protease; Peptidase S41A* | *Cytoplasmic membrane* | + |
| *SMc03791* | *Conserved hypothetical; Metallopeptidase activity* | *Cytoplasmic* | Not tested |
| *SMc03802* | *ATP-dependent protease; Peptidase S16* | *Cytoplasmic* | + |
| SMc03841 | Probable ATP-dependent CLP protease proteolytic subunit; Peptidase S14 | Cytoplasmic | Not tested |
| *SMc03872* | *Hypothetical protein; Peptidase M48* | *Unknown* | + |
| SMc03943 | Conserved hypothetical; Glutamine amidotransferase superfamily; Peptidase C26 | Cytoplasmic | Not tested |
| SMc04010 | Hypothetical protein; Hedgehog/DD-peptidase | Periplasmic | Not tested |
| SMc04012 | Peptidase M3B, oligoendopeptidase F | Cytoplasmic | + |
| SMc04031 | Putative proline iminopeptidase; Peptidase S33 | Cytoplasmic | Not tested |
| SMc04033 | Putative proline iminopeptidase; Peptidase S33 | Cytoplasmic | Not tested |
| *SMc04091* | *Putative protease transmembrane protein; Peptidase M48* | *Cytoplasmic membrane* | + |
| *SMc04113* | *Putative pilus assembly transmembrane protein; Peptidase A24A* | *Cytoplasmic membrane* | Not tested |
| SMc04198 | Phage-related repressor protein C; Peptidase S24/S26A/S26B/S26C | Cytoplasmic | Not tested |
| *SMc04217* | *Hypothetical; aspartic-type peptidase activity* | *Cytoplasmic membrane* | + |
| *SMc04226* | *Putative protease inhibitor* | *Unknown* | + |
| SMc04291 | Putative L-sorbosone dehydrogenase (sndh) protein; Peptidase M14 | Cytoplasmic membrane | Not tested |
| *SMc04352* | *Hypothetical; transglutaminase-like cysteine peptidase* | *Unknown* | + |
| *SMc04403* | *Probable Peptidyl-dipeptidase Dcp; Peptidase M3A/M3B* | *Cytoplasmic* | Not tested |
| *SMc04459* | *Probable metalloprotease transmembrane protein; Peptidase M41* | *Cytoplasmic membrane* | + |

^a^ Localization determined using PSORTb.

^b^ A “+” fixation phenotype indicates that plants were comparable in size and appearance to positive control plants, which were inoculated with *S. meliloti* C307 harboring an empty overexpression plasmid. The “+/-” fixation phenotype indicates that plants were significantly changed in size and appearance compared to positive control plants, but not as severely changed as *hrrP* or uninoculated control plants.

^c^ Italicized text indicates a peptidase has a homolog in each of the other 23 fully sequenced strains of *S. meliloti* based on an OrthoMCL analysis.


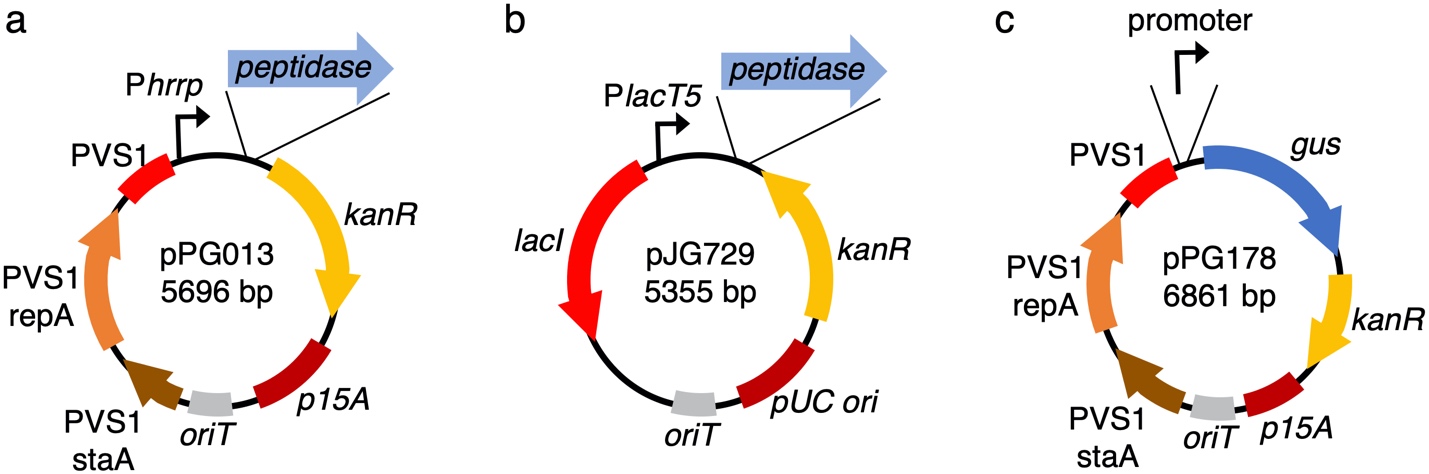


**Figure S1.** Maps of plasmids used in this study. Features of plasmids for candidate peptidase overexpression (**a**), recombinant protein expression (**b**), and promoter-*gus* analysis (**c**) are shown.

**
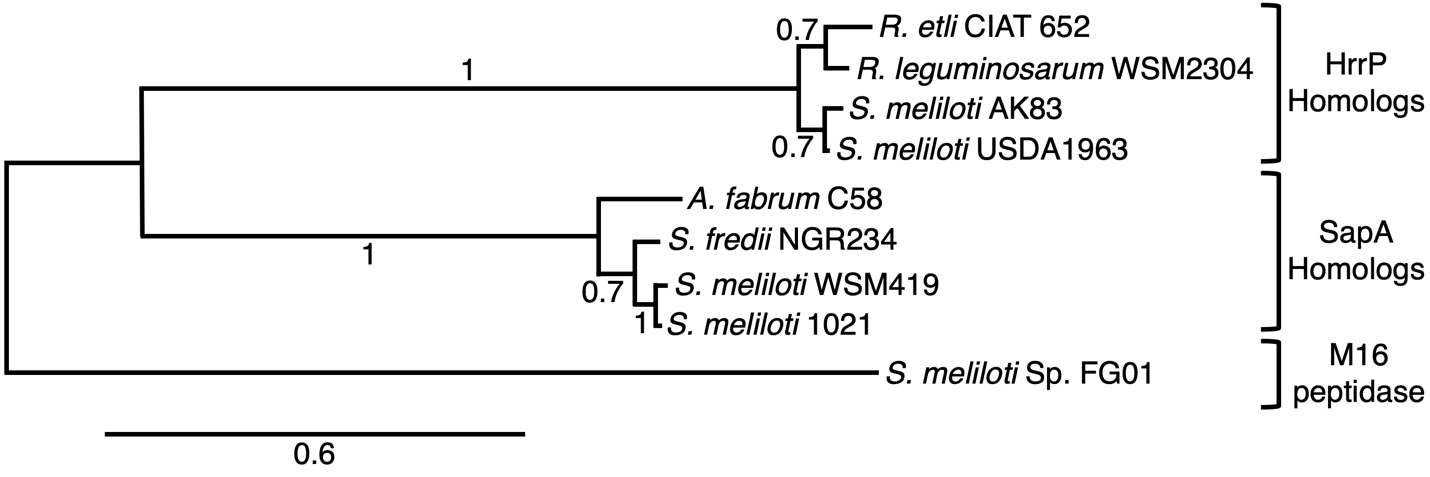
**

**Figure S2.** Phylogenetic relationships between SapA and HrrP homologs. Phylogenetic tree was reconstructed using the Bayesian inference method and rendered using TreeDyn. Branches are annotated with bootstrap values at the nodes.

**
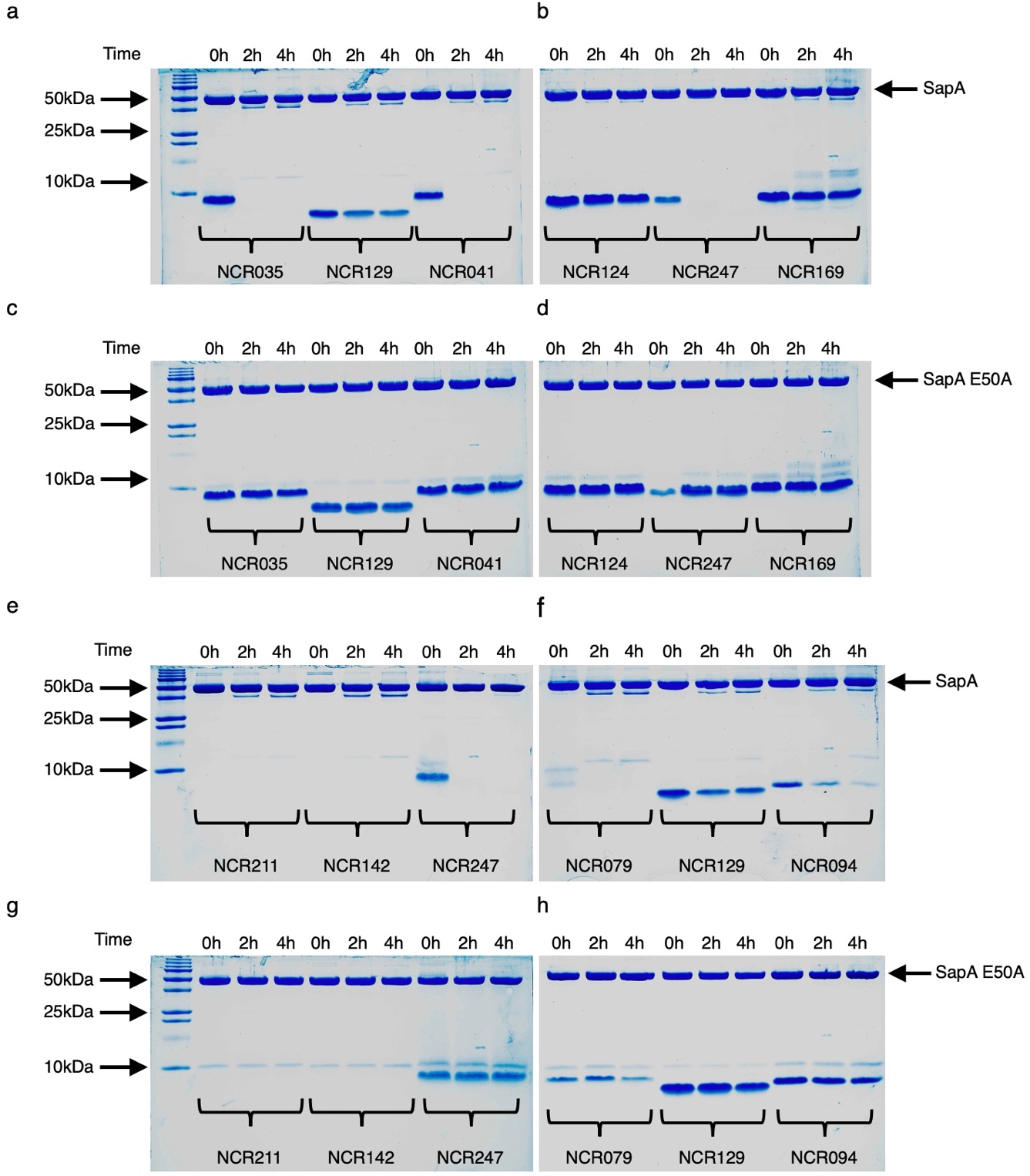
**

**Figure S3.** Full-length gel images of *in vitro* NCR peptide degradation by SapA. (**a** - **h**) SapA and a catalytically inactive version (SapA E50A) were each incubated with several different NCR peptides individually and analyzed via tricine gel at 0, 2, and 4 hours. Two experiments were run where each of 4 gels were processed in parallel. Images of representative experiments are shown above though each peptide was tested for degradation by SapA in at least three separate experiments with equivalent results. NCR211, NCR142, and NCR079 were not included in the main text due to their absence in the gels, likely from degradation of the NCR peptides in storage.

**
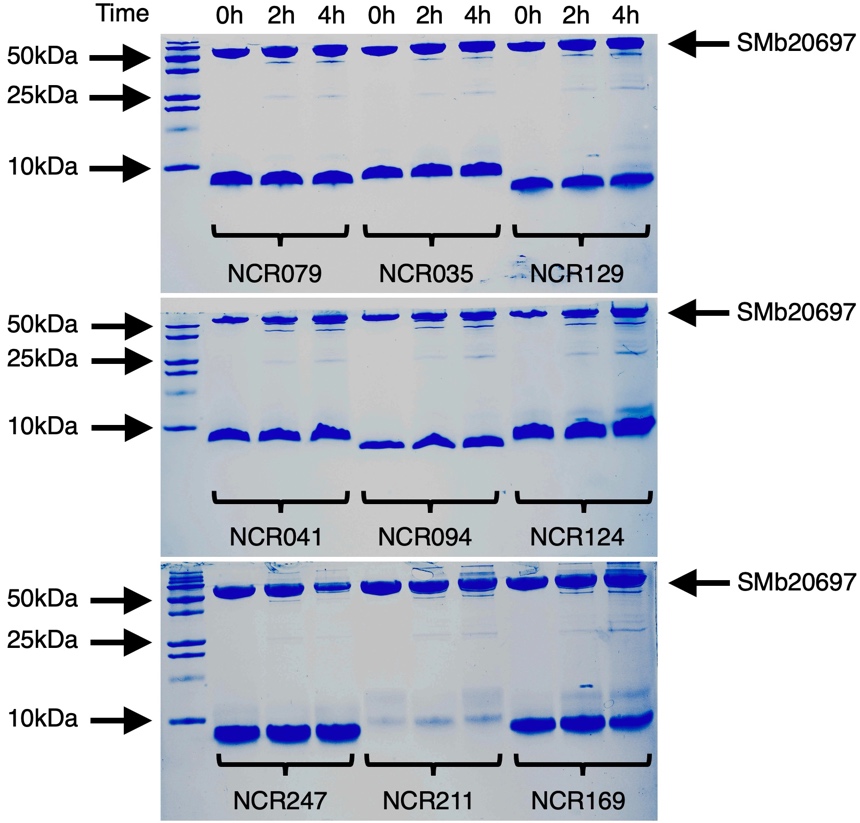
**

**Figure S4.** Treatment of NCR peptides with SMb20697 *in vitro*. SMb20697 was incubated with several different NCR peptides individually and analyzed via tricine gel at 0, 2, and 4 hours.


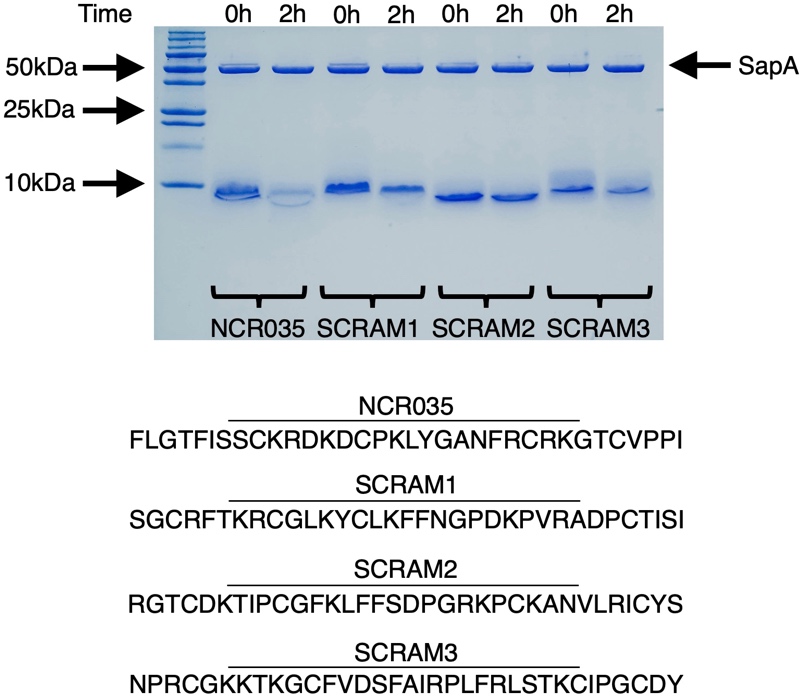


**Figure S5.** Degradation of NCR035 and scrambled variants by SapA *in vitro*. SapA was incubated with NCR035 and three variants with randomly scrambled sequences individually and analyzed via tricine gel at 0 and 2 hours. Images of representative experiments are shown above, though each peptide was tested for degradation in at least three separate experiments with equivalent results.

**Table S2.** Strains used in this study.

| **Strain name** | **Brief description** | **Reference** |
| --- | --- | --- |
| NiCO21 (DE3) | *E. coli* NiCO21 | New England Biolabs |
| B001 | *E. coli* DH5α + pRK600 conjugation plasmid, Cm^R^ | (33) |
| B100 | *S. meliloti* RM1021, Sm^R^ | (33) |
| C307 | *S. meliloti* B800 (host-restriction plasmid-cured), Sm^R^ | (33) |
| Peptidase screen | |  |
| G026 | *S. meliloti* C307 + pPG013, Sm^R^/Nm^R^ | This study |
| G027 | *S. meliloti* C307 + pPG014, Sm^R^/Nm^R^ | This study |
| G033 | *S. meliloti* C307 + pPG020, Sm^R^/Nm^R^ | This study |
| G035 | *S. meliloti* C307 + pPG022, Sm^R^/Nm^R^ | This study |
| G037 | *S. meliloti* C307 + pPG024, Sm^R^/Nm^R^ | This study |
| G043 | *S. meliloti* C307 + pPG030, Sm^R^/Nm^R^ | This study |
| G049 | *S. meliloti* C307 + pPG036, Sm^R^/Nm^R^ | This study |
| G051 | *S. meliloti* C307 + pPG038, Sm^R^/Nm^R^ | This study |
| G055 | *S. meliloti* C307 + pPG042, Sm^R^/Nm^R^ | This study |
| G057 | *S. meliloti* C307 + pPG044, Sm^R^/Nm^R^ | This study |
| G065 | *S. meliloti* C307 + pPG052, Sm^R^/Nm^R^ | This study |
| G067 | *S. meliloti* C307 + pPG054, Sm^R^/Nm^R^ | This study |
| G069 | *S. meliloti* C307 + pPG056, Sm^R^/Nm^R^ | This study |
| G073 | *S. meliloti* C307 + pPG060, Sm^R^/Nm^R^ | This study |
| G079 | *S. meliloti* C307 + pPG066, Sm^R^/Nm^R^ | This study |
| G081 | *S. meliloti* C307 + pPG068, Sm^R^/Nm^R^ | This study |
| G087 | *S. meliloti* C307 + pPG074, Sm^R^/Nm^R^ | This study |
| G091 | *S. meliloti* C307 + pPG078, Sm^R^/Nm^R^ | This study |
| G095 | *S. meliloti* C307 + pPG082, Sm^R^/Nm^R^ | This study |
| G099 | *S. meliloti* C307 + pPG086, Sm^R^/Nm^R^ | This study |
| G107 | *S. meliloti* C307 + pPG094, Sm^R^/Nm^R^ | This study |
| G109 | *S. meliloti* C307 + pPG096, Sm^R^/Nm^R^ | This study |
| AB119 | *S. meliloti* C307 + pPG100, Sm^R^/Nm^R^ | This study |
| AB120 | *S. meliloti* C307 + pPG111, Sm^R^/Nm^R^ | This study |
| AB123 | *S. meliloti* C307 + pPG116, Sm^R^/Nm^R^ | This study |
| AB124 | *S. meliloti* C307 + pPG118, Sm^R^/Nm^R^ | This study |
| AB125 | *S. meliloti* C307 + pPG120, Sm^R^/Nm^R^ | This study |
| AB128 | *S. meliloti* C307 + pPG140, Sm^R^/Nm^R^ | This study |
| AB130 | *S. meliloti* C307 + pPG148, Sm^R^/Nm^R^ | This study |
| AB131 | *S. meliloti* C307 + pPG152, Sm^R^/Nm^R^ | This study |
| Promoter-*gus* fusions | |  |
| G114 | *S. meliloti* C307 + pPG159, Sm^R^/Nm^R^ | This study |
| G116 | *S. meliloti* C307 + pPG161, Sm^R^/Nm^R^ | This study |
| G121 | *S. meliloti* C307 + pPG168, Sm^R^/Nm^R^ | This study |
| G128 | *S. meliloti* C307 + pPG178, Sm^R^/Nm^R^ | This study |

**Table S3.** Plasmids used in this study.

| **Plasmid name** | **Brief description** | **Reference** |
| --- | --- | --- |
| Peptidase screen | |  |
| pPG013 | Peptidase overexpression plasmid. Km^R^/Nm^R^ | This study |
| pPG014 | pPG013 + Hrrp, Km^R^/Nm^R^ | This study |
| pPG020 | pPG013 + *SMc03783*, Km^R^/Nm^R^ | This study |
| pPG022 | pPG013 + *SMa0142*, Km^R^/Nm^R^ | This study |
| pPG024 | pPG013 + *SMc01905*, Km^R^/Nm^R^ | This study |
| pPG030 | pPG013 + *SMb21002*, Km^R^/Nm^R^ | This study |
| pPG036 | pPG013 + *SMb20697*, Km^R^/Nm^R^ | This study |
| pPG038 | pPG013 + *SMc03286*, Km^R^/Nm^R^ | This study |
| pPG042 | pPG013 + *SMc03802*, Km^R^/Nm^R^ | This study |
| pPG044 | pPG013 + *SMc02432*, Km^R^/Nm^R^ | This study |
| pPG052 | pPG013 + *SMc01135*, Km^R^/Nm^R^ | This study |
| pPG054 | pPG013 + *SMa1329*, Km^R^/Nm^R^ | This study |
| pPG056 | pPG013 + *SMc04091*, Km^R^/Nm^R^ | This study |
| pPG060 | pPG013 + *SMc02095*, Km^R^/Nm^R^ | This study |
| pPG066 | pPG013 + *SMc01438*, Km^R^/Nm^R^ | This study |
| pPG068 | pPG013 + *SMc00451* (*sapA*), Km^R^/Nm^R^ | This study |
| pPG074 | pPG013 + *SMc03769*, Km^R^/Nm^R^ | This study |
| pPG078 | pPG013 + *SMb21495*, Km^R^/Nm^R^ | This study |
| pPG082 | pPG013 + *SMc02547*, Km^R^/Nm^R^ | This study |
| pPG086 | pPG013 + *SMc02825*, Km^R^/Nm^R^ | This study |
| pPG094 | pPG013 + *SMc00857*, Km^R^/Nm^R^ | This study |
| pPG096 | pPG013 + *SMc01524*, Km^R^/Nm^R^ | This study |
| pPG100 | pPG013 + *SMc01001*, Km^R^/Nm^R^ | This study |
| pPG111 | pPG013 + *SMc02577*, Km^R^/Nm^R^ | This study |
| pPG116 | pPG013 + *SMc03768*, Km^R^/Nm^R^ | This study |
| pPG118 | pPG013 + *SMc04352*, Km^R^/Nm^R^ | This study |
| pPG120 | pPG013 + *SMa1128*, Km^R^/Nm^R^ | This study |
| pPG140 | pPG013 + *SMb20434*, Km^R^/Nm^R^ | This study |
| pPG148 | pPG013 + *SMa1292*, Km^R^/Nm^R^ | This study |
| pPG152 | pPG013 + *SMa1126*, Km^R^/Nm^R^ | This study |
| Protein expression for peptide degradation | |  |
| pAB059 | pJG729 + SapA in NiCo21/pRARE, Km^R^/Nm^R^ | This study |
| pAB061 | pJG729 + SapA (E50A) in NiCo21/pRARE, Km^R^/Nm^R^ | This study |
| pAB062 | pJG729 + SMb20697 w/N-terminal his_6_ tag in NiCo21/pRARE, Km^R^/Nm^R^ | This study |
| pAB063 | pJG729 + SMb20697 w/C-terminal his_6_ tag in NiCo21/pRARE; Km^R^/Nm^R^ | This study |
| Promoter-*gus* fusions | |  |
| pPG159 | pPG178 + *hrrP* promoter-*gus*; Km^R^/Nm^R^ | This study |
| pPG161 | pPG178 + *sapA* promoter-*gus*; Km^R^/Nm^R^ | This study |
| pPG168 | pPG178 + *trp* promoter-*gus*; Km^R^/Nm^R^ | This study |
| pPG178 | pPG012 + *gus*; plasmid for testing free-living/nodule promoter activity; Km^R^/Nm^R^ | This study |

**Table S4.** Primers used in this study.

| **Primer name** | **Brief description** | **Sequence** |
| --- | --- | --- |
| Peptidase screen | |  |
| oPG003 | F: pPG013 check primer | ACGGCCAGGCAATCTACCAG |
| oPG004 | R: pPG013 check primer | CCAGTTTACTTTGCAGGGCTTC |
| oPG007 | F: SacI_Primer for *hrrP* promoter | CGCgagctcTCTCGTCGAGAACGT |
| oPG008 | R: EcoRI_Primer for *hrrP* promoter | CGCgaattcGACGGAATATCCGCG |
| oPG021 | F: XbaI_SMa1128 | GCGtctagaGGAGGTACTGAATGCTCAAGATATCG |
| oPG022 | R: HindIII_SMa1128 | GCGaagcttCTATTGAACATCAATGCG |
| oPG025 | F: BamHI_SMc03872 | CGCggatccGGCATGATAAGGCAGGCAGT |
| oPG026 | R: XbaI_SMc03872 | CGCtctagaGGCAGCCCCTGCGCCG |
| oPG027 | F: XbaI_SMc03783 | CGCtctagaCAGAGGTTGGAAGGGCGCGA |
| oPG028 | R: HindIII_SMc03783 | GCGaagcttAACTCTGCGGGTGAGGGGG |
| oPG029 | F: BamHI_SMa0142 | CGCggatccCTAGCCGATTGAGGTTTTCG |
| oPG030 | R: XbaI_SMa0142 | CGCtctagaACCAGACCAAACCTGCTTAA |
| oPG031 | F: XhoI_SMc01905 | CGCctcgagCATCGGGACGGAAAGGAAATG |
| oPG032 | R: XbaI_SMc01905 | CGCtctagaTACCACCCTATCTGCACTGC |
| oPG037 | F: BamHI_SMb21002 | CGCggatccGGCCGCGAAAGCGAGACATC |
| oPG038 | R: XbaI_SMb21002 | CGCtctagaGGTGCCTCGTTCGGACGAG |
| oPG043 | F: XbaI_SMb20697 | CGCtctagaAACTGAAGACGGAGAATTGC |
| oPG044 | R: HindIII_SMb20697 | CGCaagcttCTTTTTTGCCTATCTGCGAG |
| oPG045 | F: BamHI_SMc01280 | CGCggatccCAACGAAAGGGAGAATGCTT |
| oPG046 | R: XbaI_SMc01280 | CGCtctagaATGGGGGGCGAAGAGGTCG |
| oPG047 | F: BamHI_SMc03286 | CGCggatccTCAAGGATCAAGGTGAGCCG |
| oPG048 | R: XbaI_SMc03286 | CGCtctagaTGAAATATCAGTTGTCCTTTG |
| oPG051 | F: EcoRI_SMc01885 | CGCgaattcGCCAAGCTTACCGGGAAAGT |
| oPG052 | R: XhoI_SMc01885 | CGCctcgagCGGGCCGCCCGTTCCGA |
| oPG053 | F: BamHI_SMc03802 | CGCggatccGCTATCTGCAGGATCTGTCG |
| oPG054 | R: XbaI_SMc03802 | CGCtctagaCGTTGATGTCCATCGCTCTT |
| oPG055 | F: EcoRI_SMc02432 | CGCgaattcTTTAGGGGACCTCCAGCCTG |
| oPG056 | R: XhoI_SMc02432 | CGCctcgagCGCGATACCGTTGCTGTGTG |
| oPG059 | F: XbaI_SMc00539 | CGCtctagaCGCCTCCGGACGCGCAAACT |
| oPG060 | R: HindIII_SMc00539 | CGCaagcttTAGCTGCCCGCACGCCCATT |
| oPG063 | F: BamHI_SMc01135 | CGCggatccTTACCAAAAATCGGAGACAC |
| oPG064 | R: XbaI_SMc01135 | CGCtctagaCCTCGTGACGCGGCTAGCA |
| oPG065 | F: XbaI_SMa1329 | CGCtctagaACGGCTTTGTGGGAGAAATT |
| oPG066 | R: HindIII_SMa1329 | CGCaagcttTTAGGGTTCTTGGCGCCGAG |
| oPG067 | F: BamHI_SMc04091 | CGCggatccCGATTTGGTTGGAGCTCATT |
| oPG068 | R: XbaI_SMc04091 | CGCtctagaGAATCGTTCTTATTGTCTTC |
| oPG071 | F: BamHI_SMc02095 | CGCggatccGCGGACGCGTTAGGAATGAC |
| oPG072 | R: XbaI_SMc02095 | CGCtctagaCGAATCCTTAACGATGTCCG |
| oPG075 | F: BamHI_SMc04217 | CGCggatccGGCGCGAGAACGAAACGGCG |
| oPG076 | R: XbaI_SMc04217 | CGCtctagaGTAACTGAGGCTGGATCTG |
| oPG077 | F: XhoI_SMc01438 | CGCctcgagAAATGATAGGAGCCTAGCGA |
| oPG078 | R: XbaI_SMc01438 | CGCtctagaGGCCGCGAAGCGGTAGCA |
| oPG079 | F: BamHI_SMc00451 (*sapA*) | CGCggatccTAGAGCGAAAGATCACGGAT |
| oPG080 | R: XbaI_SMc00451 (*sapA*) | CGCtctagaAGGACCTTGAAGGAAGGCAT |
| oPG081 | F: BamHI_SMa0034 | CGCggatccCCTGGATGGCCTGGGAAAAA |
| oPG082 | R: XbaI_SMa0034 | CGCtctagaTTCGCTCATGGCTTTTCCAA |
| oPG085 | F: BamHI_SMc03769 | CGCggatccTGCTTTTGTGAGGGGACCGT |
| oPG086 | R: XbaI_SMc03769 | CGCtctagaCGATACCATGTGTCCGCCTC |
| oPG087 | F: BamHI_SMb21496 | CGCggatccCAGTTCGGTGCACCGCAGAC |
| oPG088 | R: XbaI_SMb21496 | CGCtctagaGCCCTTCTGCAGCGTGACGG |
| oPG089 | F: BamHI_SMb21495 | CGCggatccATTGCCTATTCGTTTCCGAT |
| oPG090 | R: XbaI_SMb21495 | CGCtctagaCCTGCAGCGCGGCAATGTCG |
| oPG091 | F: BamHI_SMc00298 | CGCggatccGCCTGAAACAACGGAAGTCT |
| oPG092 | R: XbaI_SMc00298 | CGCtctagaCGAAAAGCTCGTCGGGTTCG |
| oPG093 | F: BamHI_SMc02547 | CGCggatccGCCGCCCGAGGAAGATCTAT |
| oPG094 | R: XbaI_SMc02547 | CGCtctagaACTGATCGTTTGCGGGTCTT |
| oPG097 | F: BamHI_SMc02825 | CGCggatccATCCGCCAGTCGAGGATTCC |
| oPG098 | R: XbaI_SMc02825 | CGCtctagaTTTGCAGCTCGATTGCGCGA |
| oPG101 | F: XbaI_SMc01648 | CGCtctagaGCACCGATCGGAGGCAGCCA |
| oPG102 | R: HindIII_SMc01648 | CGCaagcttGGGCGGTCGCGCGCCGAT |
| oPG105 | F: BamHI_SMc00857 | CGCggatccATTGCAGCGGGCAGGAGTTG |
| oPG106 | R: XbaI_SMc00857 | CGCtctagaGCCCGCGCAAGCAACGGG |
| oPG111 | F: BamHI_ SMc01001 | CGCggatccGACCGAAGGGAAGAGGCAGC |
| oPG112 | R: XbaI_SMc01001 | CGCtctagaGCTGGCGGCGCCTCACCCAA |
| oPG113 | F: BamHI_SMa0095 | CGCggatccAGCGATGCAGGGAGGGGCGA |
| oPG114 | R: XbaI_SMa0095 | CGCtctagaGCCTGATCAGACCTTCACTG |
| oPG115 | F: BamHI_SMc00291 | CGCggatccACGGAGAGCGGGGAAAAGGC |
| oPG116 | R: XbaI_SMc00291 | CGCtctagaGTTTGAATGTAGCGATCGCC |
| oPG121 | F: BamHI_SMc02577 | CGCggatccGGACGGAATTTGAGAGGAATC |
| oPG122 | R: XbaI_SMc02577 | CGCtctagaGTGCCGGGTCAGAGGATCG |
| oPG127 | F: BamHI_SMc03768 | CGCggatccAACTAATCGAGAGGCGGACA |
| oPG128 | R: XbaI_SMc03768 | CGCtctagaCAAACAAGAGTTCTCTCTAA |
| oPG129 | F: BamHI_SMc04352 | CGCggatccCTACGGCGGACCGGGGGACT |
| oPG130 | R: XbaI_SMc04352 | CGCtctagaCGGGAAAAGCGCCGTTTCAG |
| oPG133 | F: BamHI_SMb20466 | CGCggatccCATGCTTGCGACAACGCATTc |
| oPG134 | R: XbaI_SMb20466 | CGCtctagaGAGTCAGAAAAGCGAGACGC |
| oPG135 | F: BamHI_SMc04010 | CGCggatccTGCTCCCTTGTCACACCCGG |
| oPG136 | R: XbaI_SMc04010 | CGCtctagaGTTTAGGCGGCAAGCGAAGG |
| oPG151 | F: BamHI_SMb20434 | CGCggatccCGGAAGAACGAGGGAAGAGA |
| oPG152 | R: XbaI_SMb20434 | CGCtctagaAAGTGGTGCTCCCTCATCCT |
| oPG157 | F: XhoI_SMc04459 | CGCctcgagCGACAGGTTCCGGGGAGTTC |
| oPG158 | R: XbaI_SMc04459 | CGCtctagaCGGCGGCATTTGATTTGCGC |
| oPG159 | F: BamHI_SMa1292 | CGCggatccAAACGCTGGAGGTAAACAGG |
| oPG160 | R: XbaI_SMa1292 | CGCtctagaCGGATGAGGAGTCCCTTGCT |
| oPG161 | F: BamHI_SMc02024 | CGCggatccCCATCGAAATACGAGGACATGT |
| oPG162 | R: XbaI_SMc02024 | CGCtctagaCGGAAGGATCGAAGCCGAAA |
| oPG163 | F: XbaI_SMa1126 | CGCtctagaCTTGGCTAGGAGTGGAATGC |
| oPG164 | R: HindIII_SMa1126 | CGCaagcttTTAGCCGCTGACGCCCGTCG |
| Protein expression for peptide degradation | |  |
| oPG173 | F: KpnI_SMb20697 (N-terminal) | CGCggtaccGGAGGTACTGAatgCACCATCACCATCACCATACGAATGCACAAGAA |
| oAB237 | F: NdeI_SMb20697 (N-terminal) | gcgCATatgggtagcCACCATCACCATCACCACACGAATG |
| oPG174 | R: HindIII_SMb20697 (N-terminal) | CGCaagcttTCAGTCCCTGTGTCTGTC |
| oAB238 | F: NdeI_SMb20697 (C-terminal) | gcgCATATGATGACGAATGCACAAGAAGAGAC |
| oAB239 | R: HindIII_SMb20697 (C-terminal) | cgcAAGCTTTCAGTGGTGATGGTGATGGTGAGATCCACCGTCCCTGTGTCTGTCGTC |
| oPG185 | F: KpnI_SMc00451 (*sapA*) (N-terminal) | CGCggtaccGGAGGTACTGAATGCACCATCACCATCACCATAAAGTTGAGTGCACC |
| oAB233 | F: NdeI_SMc00451 (*sapA*) (N-terminal) | cgcCATatgggtagcCACCATCACCATCACCATAAAG |
| oPG186 | R: HindIII_*sapA* | CGCaagcttTCAGCTCGCCGCAGCATG |
| oPG189 | F: *sapA* (E50A) mutation | GCATCTCCTGgCgCACATGGCTTTC |
| oPG190 | R: *sapA* (E50A) mutation | GAAAGCCATGTGcGcCAGGAGATGC |
| oPG167 | R: KpnI_*sapA* (E50A) mutation | CGCggtaccGGAGGTACTGAATGAGCCTACTGCTTGACAATCTC |
| Promoter-*gus* fusions | |  |
| oPG175 | F: BamHI_*gus* | GCGggatccGGAGGTACTGAATGGTCCGTCCTGTAGAA |
| oPG176 | R: XbaI _*gus* | GCGtctagaTTATTGTTTGCCTCCCTG |
| oPG183 | F: EcoRI_*sapA* promoter | CGCgaattcAATCGGCGTTTTCGTGGC |
| oPG184 | R: XhoI_*sapA* promoter | CGCctcgagCCGTGATCTTTCGCTCTA |
| oPG193 | F: SacI_*trp* promoter | CGCgagctcGGGATGTGCTGCAAGGCG |
| oPG194 | R: EcoRI_*trp* Promoter | CGCgaattcTATCAGGAAGTGCGCCACC |

**Supplementary plasmid and insert sequences.**

pPG013 – Rhizobial peptidase overexpression plasmid (5696 bp):

7…664 = *hrrP* promoter

868…1662 = *kan^R^* coding sequence

1942…2487 = p15A *oriV*

2716…2825 = RK2 *oriT*

3098…3727 = pVS1 *staA*

4160…5228 = pVS1 *repA*

5294…5488 = pVS1 *oriV*

gagctcTCTCGTCGAGAACGTAACCGTCTTGGTCAACGGCACGCAAGAGCCAATGTTTGCGGCCGCCGATGGAAATCACCACCTCGTCCAGATGCCAGATATCCTTTCGCGAAGGCCTCTTTCTGCACAACTGCCTGGCATAAGCCGTCCCGAATTTGCGACCCCATCTCCGGATCGTCTCATGGGAGACGACGATACCGCGCTCCAGCAGCATTTCCTCGACCATCCTTAGGCTCAAAGGGAACCGAAAATACAGCCACACCGCACGGGCGATGATCTGCGGTGGAAAGCGGTGGTTCTTGTAGCTTACGGCCGGACTGTTCATCCCAACCCGTTATCCACAATCGTTAAGCCGCAGACAACGTGACATCGCCATGTCAACGTTTCGTCTCGGTTCACGGCCAGATCGCCAACCTCTTTCATCTCCATCGGAAACATCTCACCGCCAACGATCATCGCCAACTCCGCGCCCAAGCCGTCACCACCTGGCGTGAAATCGCATTGTCGATTGACGCCTGAAAAGCCGACGGTACCACCCTTCAGATCCTGGCTTCCAATAAGGCGACGCCACCGCGGGAGCCTATCGGTCCGGATGACGGCTTGACCAACTCGAATCTTTATCTTACATGACGGCATAACTACGACATAACGCGGATATTCCGTC**gaattcccaCTCGAGccaGGATCCaccTCTAGAccaGTCGACaccAAGCTT**CCACAGCAAGCGAACCGGAATTGCCAGCTGGGGCGCCCTCTGGTAAGGTTGGGAAGCCCTGCAAAGTAAACTGGATGGCTTTCTTGCCGCCAAGGATCTGATGGCGCAGGGGATCAAGATCTGATCAAGAGACAGGATGAGGATCGTTTCGCatgATTGAACAAGATGGATTGCACGCAGGTTCTCCGGCCGCTTGGGTGGAGAGGCTATTCGGCTATGACTGGGCACAACAGACAATCGGCTGCTCTGATGCCGCCGTGTTCCGGCTGTCAGCGCAGGGGCGCCCGGTTCTTTTTGTCAAGACCGACCTGTCCGGTGCCCTGAATGAACTGCAGGACGAGGCAGCGCGGCTATCGTGGCTGGCCACGACGGGCGTTCCTTGCGCAGCTGTGCTCGACGTTGTCACTGAAGCGGGAAGGGACTGGCTGCTATTGGGCGAAGTGCCGGGGCAGGATCTCCTGTCATCTCACCTTGCTCCTGCCGAGAAAGTATCCATCATGGCTGATGCAATGCGGCGGCTGCATACGCTTGATCCGGCTACCTGCCCATTCGACCACCAAGCGAAACATCGCATCGAGCGAGCACGTACTCGGATGGAAGCCGGTCTTGTCGATCAGGATGATCTGGACGAAGAGCATCAGGGGCTCGCGCCAGCCGAACTGTTCGCCAGGCTCAAGGCGCGCATGCCCGACGGCGAGGATCTCGTCGTGACCCATGGCGATGCCTGCTTGCCGAATATCATGGTGGAAAATGGCCGCTTTTCTGGATTCATCGACTGTGGCCGGCTGGGTGTGGCGGACCGCTATCAGGACATAGCGTTGGCTACCCGTGATATTGCTGAAGAGCTTGGCGGCGAATGGGCTGACCGCTTCCTCGTGCTTTACGGTATCGCCGCTCCCGATTCGCAGCGCATCGCCTTCTATCGCCTTCTTGACGAGTTCTTCtgaCCCggtaccTCAGCGCTAGCGGAGTGTATACTGGCTTACTATGTTGGCACTGATGAGGGTGTCAGTGAAGTGCTTCATGTGGCAGGAGAAAAAAGGCTGCACCGGTGCGTCAGCAGAATATGTGATACAGGATATATTCCGCTTCCTCGCTCACTGACTCGCTACGCTCGGTCGTTCGACTGCGGCGAGCGGAAATGGCTTACGAACGGGGCGGAGATTTCCTGGAAGATGCCAGGAAGATACTTAACAGGGAAGTGAGAGGGCCGCGGCAAAGCCGTTTTTCCATAGGCTCCGCCCCCCTGACAAGCATCACGAAATCTGACGCTCAAATCAGTGGTGGCGAAACCCGACAGGACTATAAAGATACCAGGCGTTTCCCCCTGGCGGCTCCCTCGTGCGCTCTCCTGTTCCTGCCTTTCGGTTTACCGGTGTCATTCCGCTGTTATGGCCGCGTTTGTCTCATTCCACGCCTGACACTCAGTTCCGGGTAGGCAGTTCGCTCCAAGCTGGACTGTATGCACGAACCCCCCGTTCAGTCCGACCGCTGCGCCTTATCCGGTAACTATCGTCTTGAGTCCAACCCGGAAAGACATGCAAAAGCACCACTGGCAGCAGCCACTGGTAATTGATTTAGAGGAGTTAGTCTTGAAGTCATGCGCCGGTTAAGGCTAAACTGAAAGGACAAGTTTTGGTGACTGCGCTCCTCCAAGCCAGTTACCTCGGTTCAAAGAGTTGGTAGCTCAGAGAACCTTCGAAAAACCGCCCTGCAAGGCGGTTTTTTCGTTTTCAGAGCAAGAGATTACGCGCAGACCAAAACGATCTCAAGAAGATCATCTTATTAAGGGGTCTGACGctcagtggaacgaaaactcacgTTAAGGGATTTTGGTCATGAGATTATCAAAAAGGATCTTCACCTAGATCCTTTTAAATTAAAAATGAAGTTTTAAATCAATCTAAAGTATATATGAGTAAACTTGGTCTGACAGTTACCAATGCTTAATCAGactAGAGCTTccatccgcttgccctcatctgttacgccggcggtagccggccagcctcgcagagcaggattcccgttgagcaccgccaggtgcgaataagggacagtgaagaaggaacacccgctcgcgggtgggcctacttcacctatcctgcccggctgacgccgttggatacaccaaggaaagtctacacgaaccctttggcaaaatcctgtatatcgtgcgAATTGAtccACCGTGCGGCTGCATGAAATCCTGGCCGGTTTGTCTGATGCCAAGCTGGCGGCCTGGCCGGCCAGCTTGGCCGCTGAAGAAACCGAGCGCCGCCGTCTAAAAAGGTGATGTGTATTTGAGTAAAACAGCTTGCGTCATGCGGTCGCTGCGTATATGATGCGATGAGTAAATAAACAAATACGCAAGGGGAACGCatgAAGGTTATCGCTGTACTTAACCAGAAAGGCGGGTCAGGCAAGACgaccatcgcaacccatctagcCCGCGCCCTGCAACTCGCCGGGGCCGATGTTCTGTTAGTCGATTCCGATCCCCAGGGCAGTGCCCGCGATTGGGCGGCCGTGCGGGAAGATCAACCGCTAACCGTTGTCGGCATCGACCGCCCGACGATTGACCGCGACGTGAAGGCCATCGGCCGGCGCGACTTCGTAGTGATCGACGGAGCGCCCCAGGCGGCGGACTTGGCTGTGTCCGCGATCAAGGCAGCCGACTTCGTGCTGATTCCGGTGCAGCCAAGCCCTTACGACATATGGGCCACCGCCGACCTGGTGGAGCTGGTTAAGCAGCGCATTGAGGTCACGGATGGAAGGCTACAAGCGGCCTTTGTCGTGTCGCGGGCGATCAAAGGCACGCGCATCGGCGGTGAGGTTGCCGAGGCGCTGGCCGGGTACGAGCTGCCCATTCTTGAGTCCCGTATCACGCAGCGCGTGAGCTACCCAGGCACTGCCGCCGCCGGCACAACCGTTCTTGAATCAGAACCCGAGGGCGACGCTGCCCGCGAGGTCCAGGCGCTGGCCGCTGAAATTAAATCAAAACTCATTtgaGTTAATGAGGTAAAGAGAAAATGAGCAAAAGCACAAACACGCTAAGTGCCGGCCGTCCGAGCGCACGCAGCAGCAAGGCTGCAACGTTGGCCAGCCTGGCAGACACGCCAGCCATGAAGCGGGTCAACTTTCAGTTGCCGGCGGAGGATCACACCAAGCTGAAGATGTACGCGGTACGCCAAGGCAAGACCATTACCGAGCTGCTATCTGAATACATCGCGCAGCTACCAGAGTAAATGAGCAAATGAATAAATGAGTAGATGAATTTTAGCGGCTAAAGGAGGCGGCATGGAAAATCAAGAACAACCAGGCACCGACGCCGTGGAATGCCCCATGTGTGGAGGAACGGGCGGTTGGCCAGGCGTAAGCGGCTGGGTTGTCTGCCGGCCCTGCAatgGCACTGGAACCCCCAAGCCCGAGGAATCGGCGTGACGGTCGCAAACCATCCGGCCCGGTACAAATCGGCGCGGCGCTGGGTGATGACCTGGTGGAGAAGTTGAAGGCCGCGCAGGCCGCCCAGCGGCAACGCATCGAGGCAGAAGCACGCCCCGGTGAATCGTGGCAAGCGGCCGCTGATCGAATCCGCAAAGAATCCCGGCAACCGCCGGCAGCCGGTGCGCCGTCGATTAGGAAGCCGCCCAAGGGCGACGAGCAACCAGATTTTTTCGTTCCGATGCTCTATGACGTGGGCACCCGCGATAGTCGCAGCATCATGGACGTGGCCGTTTTCCGTCTGTCGAAGCGTGACCGACGAGCTGGCGAGGTGATCCGCTACGAGCTTCCAGACGGGCACGTAGAGGTTTCCGCAGGGCCGGCCGGCATGGCCAGTGTGTGGGATTACGACCTGGTACTGATGGCGGTTTCCCATCTAACCGAATCCATGAACCGATACCGGGAAGGGAAGGGAGACAAGCCCGGCCGCGTGTTCCGTCCACACGTTGCGGACGTACTCAAGTTCTGCCGGCGAGCCGATGGCGGAAAGCAGAAAGACGACCTGGTAGAAACCTGCATTCGGTTAAACACCACGCACGTTGCCATGCAGCGTACGAAGAAGGCCAAGAACGGCCGCCTGGTGACGGTATCCGAGGGTGAAGCCTTGATTAGCCGCTACAAGATCGTAAAGAGCGAAACCGGGCGGCCGGAGTACATCGAGATCGAGCTAGCTGATTGGATGTACCGCGAGATCACAGAAGGCAAGAACCCGGACGTGCTGACGGTTCACCCCGATTACTTTTTGATCGATCCCGGCATCGGCCGTTTTCTCTACCGCCTGGCACGCCGCGCCGCAGGCAAGGCAGAAGCCAGATGGTTGTTCAAGACGATCTACGAACGCAGTGGCAGCGCCGGAGAGTTCAAGAAGTTCTGTTTCACCGTGCGCAAGCTGATCGGGTCAAATGACCTGCCGGAGTACGATTTGAAGGAGGAGGCGGGGCAGGCTGGCCCGATCCTAGTCATGCGCTACCGCAACCTGATCGAGGGCGAAGCATCCGCCGGTTCCtaaTGTACGGAGCAGATGCTAGGGCAAATTGCCCTAGCAGGGGAAAAAGGTCGAAAAGGTCTCTTTCCTGTGGATAGCACGTACATTGGGAACCCAAAGCCGTACATTGGGAACCGGAACCCGTACATTGGGAACCCAAAGCCGTACATTGGGAACCGGTCACACATGTAAGTGACTGATATAAAAGAGAAAAAAGGCGATTTTTCCGCCTAAAACTCTTTAAAACTTATTAAAACTCTTAAAACCCGCCTGGCCTGTGCATAACTGTCTGGCCAGCGCACAGCCGAAGAGCTGCAAAAAGCGCCTACCCTTCGGTCGCTGCGCTCCCTACGCCCCGCCGCTTCGCGTCGGCCTATCGCGGCCGCTGGCCGCTCAAAAATGGCTGGCCTacggccaggcaatctaccagGGCGCGGACAAGCCGCGCCGTCGCCACTCGACCGCCGGCGCCCACATCAAGGCACCCTGCCT

pJG729 – *E. coli* protein expression plasmid (5355 bp):

559…1374 = *kan^R^* coding sequence

1473…2131 = ColE1 *oriV*

2270…2409 = *oriT*

3511…4593 = *lacI*

4594…4671 = *lacI* promoter

4989…5033 = T5 promoter

4948…4964 = lac operator

5039…5058 = symmetric lac operator

tggcgaatgggacgcgccctgtagcggcgcattaagcgcggcgggtgtggtggttacgcgcagcgtgaccgctacacttgccagcgccctagcgcccgctcctttcgctttcttcccttcctttctcgccacgttcgccggctttccccgtcaagctctaaatcgggggctccctttagggttccgatttagtgctttacggcacctcgaccccaaaaaacttgattagggtgatggttcacgtagtgggccatcgccctgatagacggtttttcgccctttgacgttggagtccacgttctttaatagtggactcttgttccaaactggaacaacactcaaccctatctcggtctattcttttgatttataagggattttgccgatttcggcctattggttaaaaaatgagctgatttaacaaaaatttaacgcgaattttaacaaaaTATTAACGCTTACAATTTAGGTGGcacttttcggggaaatgtgcgcggaacccctatttgtttatttttctaaatacattcaaatatgtatccgctcatgaattaattcttagaaaaactcatcgagcatcaaatgaaactgcaatttattcatatcaggattatcaataccatatttttgaaaaagccgtttctgtaatgaaggagaaaactcaccgaggcagttccataggatggcaagatcctggtatcggtctgcgattccgactcgtccaacatcaatacaacctattaatttcccctcgtcaaaaataaggttatcaagtgagaaatcaccatgagtgacgactgaatccggtgagaatggcaaaagtttatgcatttctttccagacttgttcaacaggccagccattacgctcgtcatcaaaatcactcgcatcaaccaaaccgttattcattcgtgattgcgcctgagcgagacgaaatacgcgatcgctgttaaaaggacaattacaaacaggaatcgaatgcaaccggcgcaggaacactgccagcgcatcaacaatattttcacctgaatcaggatattcttctaatacctggaatgctgttttcccggggatcgcagtggtgagtaaccatgcatcatcaggagtacggataaaatgcttgatggtcggaagaggcataaattccgtcagccagtttagtctgaccatctcatctgtaacatcattggcaacgctacctttgccatgtttcagaaacaactctggcgcatcgggcttcccatacaatcgatagattgtcgcacctgattgcccgacattatcgcgagcccatttatacccatataaatcagcatccatgttggaatttaatcgcggcctagagcaagacgtttcccgttgaatatggctcataacaccccttgtattactgtttatgtaagcagacagttttattgttcatgaccaaaatcccttaacgtgagttttcgttccactgagcgtcagaccccgtagaaaagatcaaaggatcttcttgagatcctttttttctgcgcgtaatctgctgcttgcaaacaaaaaaaccaccgctaccagcggtggtttgtttgccggatcaagagctaccaactctttttccgaaggtaactggcttcagcagagcgcagataccaaatactgtccttctagtgtagccgtagttaggccaccacttcaagaactctgtagcaccgcctacatacctcgctctgctaatcctgttaccagtggctgctgccagtggcgataagtcgtgtcttaccgggttggactcaagacgatagttaccggataaggcgcagcggtcgggctgaacggggggttcgtgcacacagcccagcttggagcgaacgacctacaccgaactgagatacctacagcgtgagctatgagaaagcgccacgcttcccgaagggagaaaggcggacaggtatccggtaagcggcagggtcggaacaggagagcgcacgagggagcttccagggggaaacgcctggtatctttatagtcctgtcgggtttcgccacctctgacttgagcgtcgatttttgtgatgctcgtcaggggggcggagcctatggaaaaacgccagcaacgcggcctttttacggttcctggccttttgctggccttttgctcacatgttctttcctgcgttatcccctgattctgtggataaccgtattaccgcctttgagtgagctgataccgctcgccgcagccgaacgaccgagcgcagcgagtcagtgagcgaggaagcggaagagcgcctgatgcggtattttctccttacgcatctgtgcggtatttcacaccgcaatggtgcactctcagtacaatctgctctgatgccgcatagttaagccagtatacactccgctatcgctacgtgactgggtcatggctgcgccccgacacccgccaacacccgctgacgcgccctgacgggcttgtctgctcccggcatccgcttacagacaagctgtgaccgtctccgggagctgcatgtgtcagaggttttcaccgtcatcaccgaaacgcgcgaggcagctgcggtaaagctcatcagcgtggtcgtgaagcgattcacagatgtctgcctgttcatccgcgtccagctcgttgagtttctccagaagcgttaatgtctggcttctgataaagcgggccatgttaagggcggttttttcctgtttggtcactgatgcctccgtgtaagggggatttctgttcatgggggtaatgataccgatgaaacgagagaggatgctcacgatacgggttactgatgatgaacatgcccggttactggaacgttgtgagggtaaacaactggcggtatggatgcggcgggaccagagaaaaatcactcagggtcaatgccagcgcttcgttaatacagatgtaggtgttccacagggtagccagcagcatcctgcgatgcagatccggaacataatggtgcagggcgctgacttccgcgtttccagactttacgaaacacggaaaccgaagaccattcatgttgttgctcaggtcgcagacgttttgcagcagcagtcgcttcacgttcgctcgcgtatcggtgattcattctgctaaccagtaaggcaaccccgccagcctagccgggtcctcaacgacaggagcacgatcatgcgcacccgtggggccgccatgccggcgataatggcctgcttctcgccgaaacgtttggtggcgggaccagtgacgaaggcttgagcgagggcgtgcaagattccgaataccgcaagcgacaggccgatcatcgtcgcgctccagcgaaagcggtcctcgccgaaaatgacccagagcgctgccggcacctgtcctacgagttgcatgataaagaagacagtcataagtgcggcgacgatagtcatgccccgcgcccaccggaaggagctgactgggttgaaggctctcaagggcatcggtcgagatcccggtgcctaatgagtgagctaacttacattaattgcgttgcgctcactgcccgctttccagtcgggaaacctgtcgtgccagctgcattaatgaatcggccaacgcgcggggagaggcggtttgcgtattgggcgccagggtggtttttcttttcaccagtgagacgggcaacagctgattgcccttcaccgcctggccctgagagagttgcagcaagcggtccacgctggtttgccccagcaggcgaaaatcctgtttgatggtggttaacggcgggatataacatgagctgtcttcggtatcgtcgtatcccactaccgagatgtccgcaccaacgcgcagcccggactcggtaatggcgcgcattgcgcccagcgccatctgatcgttggcaaccagcatcgcagtgggaacgatgccctcattcagcatttgcatggtttgttgaaaaccggacatggcactccagtcgccttcccgttccgctatcggctgaatttgattgcgagtgagatatttatgccagccagccagacgcagacgcgccgagacagaacttaatgggcccgctaacagcgcgatttgctggtgacccaatgcgaccagatgctccacgcccagtcgcgtaccgtcttcatgggagaaaataatactgttgatgggtgtctggtcagagacatcaagaaataacgccggaacattagtgcaggcagcttccacagcaatggcatcctggtcatccagcggatagttaatgatcagcccactgacgcgttgcgcgagaagattgtgcaccgccgctttacaggcttcgacgccgcttcgttctaccatcgacaccaccacgctggcacccagttgatcggcgcgagatttaatcgccgcgacaatttgcgacggcgcgtgcagggccagactggaggtggcaacgccaatcagcaacgactgtttgcccgccagttgttgtgccacgcggttgggaatgtaattcagctccgccatcgccgcttccactttttcccgcgttttcgcagaaacgtggctggcctggttcaccacgcgggaaacggtctgataagagacaccggcatactctgcgacatcgtataacgttactggtttcacattcaccaccctgaattgactctcttccgggcgctatcatgccataccgcgaaaggttttgcgccattcgatggtgtccgggatctcgacgctctcccttatgcgactcctgcattaggaagcagcccagtagtaggttgaggccgttgagcaccgccgccgcaaggaatggtgcatgcaaggagatggcgcccaacagtcccccggccacggggcctgccaccatacccacgccgaaacaagcgctcatgagcccgaagtggcgagcccgatcttccccatcggtgatgtcggcgatataggcgccagcaaccgcacctgtggcgccggtgagatctcgatcccgcgaaatAATTGTGAGCGGATAACAATTacgagcttcatgcacagtgaaatcatgaaaaatttatTTGCTTtgtgagcggataacaatTATAATatgtggAATTGTGAGCGCTCACAATTccacaacggtTCTAGAaataattttgtttaactttaagaaggagatataCATatg**GGATCCaccGAATTCccaGTCGACaccAAGCTTccaCTGCAGccaGAGCTC**ggctgctaacaaagcccgaaaggaagctgagttggctgctgccaccgctgctggttcgctcataagtaaaaaacggcacctggtgccgtttttttgtctgaaacaagctgagcaataactagcataaccccttggggcctctaaacgggtcttgaggggttttttgctgaaaggaggaactatatccggat

pAB059 insert (NdeI – MGS his_6_ tag – *sapA* – HindIII):

CATatgGGTAGCCACCATCACCATCACCATaaagttgagtgcacccggctcccttccgggctgacggtggttaccgagcgaatgccgcatctggaaagcgtggcgctcggagtctggatcaagtccggttcgcgcaacgaaaccgtgaatgaacacggaattgcgcatctcctgGAGcacatggctttcaagggcacgaggcggcgcagcgcccgccagatcgccgaggaaatcgagaatgtgggcggcgaggtcaacgccgccacctcgaccgaaacgacttcctactatgcccgcgtactcaaggaccatctgccgctggcggtcgatatcctcgccgacattctgacggaatccaccttcgaggcggacgagctgcgtcgtgagaagcaggtcatcctgcaggagatcggcgcggcggacgacacgccggacgacgtcgtcttcgatcgttttgccgagaccgcctatcgtggccagacggtcggcaggccgatcctcggcacgccggaaacggtgatgtccttcagcgcggatcagatccgccagtatctcggccgcaactatacgaccgaccgcaccttcatcgtagccgccggcgccgtcgatcacgacaccatcgtgcgccaggtggaggagcgtttcgcctccctgcccgccgaacccgtttgcgctcccgtcatcgagaccgcgcgctacaccggcggcgacagccgcgagagccgcgacctgatggacgcgcaggttctgctcggcttcgaaggcaaggcctatcacgcccgtgatttctattgttcgcagatccttgccaacatcctcggcggcggcatgtcgtcccgcctgttccaggaagtgcgcgaacacaggggcctctgttactcggtctacgccttccattggggcttttccgataccggcatcttcggcgtgcatgcggcaacgggcggagaaaacctgccggagctgatgccggtgatcgtcgatgaattgcgcaagtcgtccttgagcatcgatcagcaggagatcgagcgtgcccgcgcacagatccgggcgcaattgctgatgggccaggaaagcccggccgcgcgtgcgggacaaatcgcacggcagatgatgctgtacggccggccgatccccaatgaggaactgatggaacgcctgtcgggcatcaccatcgagcgcctcaccgacctggccggccgcctgttcttcgacacggttccgacgttgtcggccatcggtccgctcggacagctggctcccctgaacgacatattgtcctcgctgaccacgaaggcggacgcgatacatgctgcggcgagctgaAAGCTT

pAB061 insert (NdeI – MGS his_6_ tag – *sapA* (E50A) – HindIII):

CATatgGGTAGCCACCATCACCATCACCATaaagttgagtgcacccggctcccttccgggctgacggtggttaccgagcgaatgccgcatctggaaagcgtggcgctcggagtctggatcaagtccggttcgcgcaacgaaaccgtgaatgaacacggaattgcgcatctcctgGCGcacatggctttcaagggcacgaggcggcgcagcgcccgccagatcgccgaggaaatcgagaatgtgggcggcgaggtcaacgccgccacctcgaccgaaacgacttcctactatgcccgcgtactcaaggaccatctgccgctggcggtcgatatcctcgccgacattctgacggaatccaccttcgaggcggacgagctgcgtcgtgagaagcaggtcatcctgcaggagatcggcgcggcggacgacacgccggacgacgtcgtcttcgatcgttttgccgagaccgcctatcgtggccagacggtcggcaggccgatcctcggcacgccggaaacggtgatgtccttcagcgcggatcagatccgccagtatctcggccgcaactatacgaccgaccgcaccttcatcgtagccgccggcgccgtcgatcacgacaccatcgtgcgccaggtggaggagcgtttcgcctccctgcccgccgaacccgtttgcgctcccgtcatcgagaccgcgcgctacaccggcggcgacagccgcgagagccgcgacctgatggacgcgcaggttctgctcggcttcgaaggcaaggcctatcacgcccgtgatttctattgttcgcagatccttgccaacatcctcggcggcggcatgtcgtcccgcctgttccaggaagtgcgcgaacacaggggcctctgttactcggtctacgccttccattggggcttttccgataccggcatcttcggcgtgcatgcggcaacgggcggagaaaacctgccggagctgatgccggtgatcgtcgatgaattgcgcaagtcgtccttgagcatcgatcagcaggagatcgagcgtgcccgcgcacagatccgggcgcaattgctgatgggccaggaaagcccggccgcgcgtgcgggacaaatcgcacggcagatgatgctgtacggccggccgatccccaatgaggaactgatggaacgcctgtcgggcatcaccatcgagcgcctcaccgacctggccggccgcctgttcttcgacacggttccgacgttgtcggccatcggtccgctcggacagctggctcccctgaacgacatattgtcctcgctgaccacgaaggcggacgcgatacatgctgcggcgagctgaAAGCTT

pAB062 insert (NdeI – MGS his_6_ tag – *SMb20697* – HindIII):

CATatgGGTAGCCACCATCACCATCACCACacgaatgcacaagaagagactgtctctcgcgctggaagccggatcgacagcgagcgcgtccggcaattcgctctgcgcatgacgtcctggcccagtgaaaccggcacgccgggcgaagcatccttcgcggatcgcctccatgggcttctcggcgaactcccctattttcgggagcatccgcaggacctgggcctccttgcaagtcacggcgaaccgctgacccgcaatgtcgttgcgctcgttcgcggcacgggcaagcgaacactggtcatggccggccacttcgacaccgtgtcgaccgacaactatcacgagctcaaggcgctggcatgcgacagcctggcgctcaaggatgcactcatcgaaagcctgtcggcgcgaaccggccgatccgaacaggaagagcgggccctgcaggacctggcgagtggcgacttcctccccggccgcggcctgctcgacatgaagagcggactcgccgtggccatagcctgtctcgaacaattcgcggccgacacggaccgacagggcaatctcatgctggtcgccacccccgacgaggaacgggaaagccggggaatgcgatcgctccgggacgcgttgcccggcttggtcagggatttcgacatcgaaatagccgggggcatcaacctcgacgtgacctcggatcagggcgacggcagcgaagggcgggccgtttacgccggcacgatcggcaagctcctaccctttgccctggtgatcggctgcagctctcatgcgagctaccccttcgaaggggtgagcgcacaggccatggcggccgggatcctggcacgcctggaagggaatgcttccctggcggatcgcgacgacaacgacatctcgccgccgccgatctgcctcgaggcgaaggatctgcgcgacggttacgaagtgacgacgccggagcgcttctggatagctttcaactggctctaccattcgatgacggcggacgcactctttgagcgcttccgagaggaggtgctgaccggcgcgaacgaggccatcgagacgtttgcggcacagtcggcggaatacggcaagctcgtcggcagaagtgcgggcggcctaccagccaagccgcgccttctgtcgttccaggaattgcgggcggcggctgcacgcgttttcgggcacggcttcgacgcattctatgccgagaaggaaagggaattcgcccagagcgacaacccgctcgtcgctacgcggcaacttactgagtggctcgtcggtatcgcgcgcctttccggccccgccatcgtcatcggcttctcgggattgcattatccgcccagccatctgcgcctggcggaaggaaacgaccgctcccttcatcaggcgatcgagaaggcgcgtgccggtcttggcaacgatcccgcgcgaagcctcatctggaagccgcatttttacggaatctccgacatgagtttcctcgggcttgccgccagcggcagccaggtcgtttcggacaacaccccgatctcacgtctggtcgatcggccatccgaaaacgcgctgcgttttccgaccgtcaatctcggaccttggggacgggagttccaccagaagttcgagcgcgtccatgaaccctacgcgttcagggtcctcccggagctcgtttccgaaatcgccaggaccttcctcggcgacgacagacacagggactgaAAGCTT

pAB063 insert (NdeI – *SMb20697* – GGS his_6_ tag – HindIII):

CATATGatgacgaatgcacaagaagagactgtctctcgcgctggaagccggatcgacagcgagcgcgtccggcaattcgctctgcgcatgacgtcctggcccagtgaaaccggcacgccgggcgaagcatccttcgcggatcgcctccatgggcttctcggcgaactcccctattttcgggagcatccgcaggacctgggcctccttgcaagtcacggcgaaccgctgacccgcaatgtcgttgcgctcgttcgcggcacgggcaagcgaacactggtcatggccggccacttcgacaccgtgtcgaccgacaactatcacgagctcaaggcgctggcatgcgacagcctggcgctcaaggatgcactcatcgaaagcctgtcggcgcgaaccggccgatccgaacaggaagagcgggccctgcaggacctggcgagtggcgacttcctccccggccgcggcctgctcgacatgaagagcggactcgccgtggccatagcctgtctcgaacaattcgcggccgacacggaccgacagggcaatctcatgctggtcgccacccccgacgaggaacgggaaagccggggaatgcgatcgctccgggacgcgttgcccggcttggtcagggatttcgacatcgaaatagccgggggcatcaacctcgacgtgacctcggatcagggcgacggcagcgaagggcgggccgtttacgccggcacgatcggcaagctcctaccctttgccctggtgatcggctgcagctctcatgcgagctaccccttcgaaggggtgagcgcacaggccatggcggccgggatcctggcacgcctggaagggaatgcttccctggcggatcgcgacgacaacgacatctcgccgccgccgatctgcctcgaggcgaaggatctgcgcgacggttacgaagtgacgacgccggagcgcttctggatagctttcaactggctctaccattcgatgacggcggacgcactctttgagcgcttccgagaggaggtgctgaccggcgcgaacgaggccatcgagacgtttgcggcacagtcggcggaatacggcaagctcgtcggcagaagtgcgggcggcctaccagccaagccgcgccttctgtcgttccaggaattgcgggcggcggctgcacgcgttttcgggcacggcttcgacgcattctatgccgagaaggaaagggaattcgcccagagcgacaacccgctcgtcgctacgcggcaacttactgagtggctcgtcggtatcgcgcgcctttccggccccgccatcgtcatcggcttctcgggattgcattatccgcccagccatctgcgcctggcggaaggaaacgaccgctcccttcatcaggcgatcgagaaggcgcgtgccggtcttggcaacgatcccgcgcgaagcctcatctggaagccgcatttttacggaatctccgacatgagtttcctcgggcttgccgccagcggcagccaggtcgtttcggacaacaccccgatctcacgtctggtcgatcggccatccgaaaacgcgctgcgttttccgaccgtcaatctcggaccttggggacgggagttccaccagaagttcgagcgcgtccatgaaccctacgcgttcagggtcctcccggagctcgtttccgaaatcgccaggaccttcctcggcgacgacagacacagggacGGTGGATCTCACCATCACCATCACCACtgaAAGCTT

pPG178 – Rhizobial *gus* expression plasmid (6861 bp):

45…1856 = *gus* coding sequence

2033…2827 = *kan^R^* coding sequence

3107…3652 = p15A *oriV*

3845…4059 = RK2 *oriT*

4263…4892 = pVS1 *staA*

5287…6393 = pVS1 *repA*

6459…6653 = pVS1 *oriV*

**GAGCTCaccGAATTCccaCTCGAGccaGGATCC**ggaggtactgaatggtccgtcctgtagaaaccccaacccgtgaaatcaaaaaactcgacggcctgtgggcattcagtctggatcgcgaaaactgtggaattgatcagcgttggtgggaaagcgcgttacaagaaagccgggcaattgctgtgccaggcagttttaacgatcagttcgccgatgcagatattcgtaattatgcgggcaacgtctggtatcagcgcgaagtctttataccgaaaggttgggcaggccagcgtatcgtgctgcgtttcgatgcggtcactcattacggcaaagtgtgggtcaataatcaggaagtgatggagcatcagggcggctatacgccatttgaagccgatgtcacgccgtatgttattgccgggaaaagtgtacgtatcaccgtttgtgtgaacaacgaactgaactggcagactatcccgccgggaatggtgattaccgacgaaaacggcaagaaaaagcagtcttacttccatgatttctttaactatgccggaatccatcgcagcgtaatgctctacaccacgccgaacacctgggtggacgatatcaccgtggtgacgcatgtcgcgcaagactgtaaccacgcgtctgttgactggcaggtggtggccaatggtgatgtcagcgttgaactgcgtgatgcggatcaacaggtggttgcaactggacaaggcactagcgggactttgcaagtggtgaatccgcacctctggcaaccgggtgaaggttatctctatgaactgtgcgtcacagccaaaagccagacagagtgtgatatctacccgcttcgcgtcggcatccggtcagtggcagtgaagggcgaacagttcctgattaaccacaaaccgttctactttactggctttggtcgtcatgaagatgcggacttgcgtggcaaaggattcgataacgtgctgatggtgcacgaccacgcattaatggactggattggggccaactcctaccgtacctcgcattacccttacgctgaagagatgctcgactgggcagatgaacatggcatcgtggtgattgatgaaactgctgctgtcggctttaacctctctttaggcattggtttcgaagcgggcaacaagccgaaagaactgtacagcgaagaggcagtcaacggggaaactcagcaagcgcacttacaggcgattaaagagctgatagcgcgtgacaaaaaccacccaagcgtggtgatgtggagtattgccaacgaaccggatacccgtccgcaaggtgcacgggaatatttcgcgccactggcggaagcaacgcgtaaactcgacccgacgcgtccgatcacctgcgtcaatgtaatgttctgcgacgctcacaccgataccatcagcgatctctttgatgtgctgtgcctgaaccgttattacggatggtatgtccaaagcggcgatttggaaacggcagagaaggtactggaaaaagaacttctggcctggcaggagaaactgcatcagccgattatcatcaccgaatacggcgtggatacgttagccgggctgcactcaatgtacaccgacatgtggagtgaagagtatcagtgtgcatggctggatatgtatcaccgcgtctttgatcgcgtcagcgccgtcgtcggtgaacaggtatggaatttcgccgattttgcgacctcgcaaggcatattgcgcgttggcggtaacaagaaagggatcttcactcgcgaccgcaaaccgaagtcggcggcttttctgctgcaaaaacgctggactggcatgaacttcggtgaaaaaccgcagcagggaggcaaacaataaTCTAGAccaGTCGACaccAAGCTTCCACAGCAAGCGAACCGGAATTGCCAGCTGGGGCGCCCTCTGGTAAGGTTGGGAAGCCCTGCAAAGTAAACTGGATGGCTTTCTTGCCGCCAAGGATCTGATGGCGCAGGGGATCAAGATCTGATCAAGAGACAGGATGAGGATCGTTTCGCatgATTGAACAAGATGGATTGCACGCAGGTTCTCCGGCCGCTTGGGTGGAGAGGCTATTCGGCTATGACTGGGCACAACAGACAATCGGCTGCTCTGATGCCGCCGTGTTCCGGCTGTCAGCGCAGGGGCGCCCGGTTCTTTTTGTCAAGACCGACCTGTCCGGTGCCCTGAATGAACTGCAGGACGAGGCAGCGCGGCTATCGTGGCTGGCCACGACGGGCGTTCCTTGCGCAGCTGTGCTCGACGTTGTCACTGAAGCGGGAAGGGACTGGCTGCTATTGGGCGAAGTGCCGGGGCAGGATCTCCTGTCATCTCACCTTGCTCCTGCCGAGAAAGTATCCATCATGGCTGATGCAATGCGGCGGCTGCATACGCTTGATCCGGCTACCTGCCCATTCGACCACCAAGCGAAACATCGCATCGAGCGAGCACGTACTCGGATGGAAGCCGGTCTTGTCGATCAGGATGATCTGGACGAAGAGCATCAGGGGCTCGCGCCAGCCGAACTGTTCGCCAGGCTCAAGGCGCGCATGCCCGACGGCGAGGATCTCGTCGTGACCCATGGCGATGCCTGCTTGCCGAATATCATGGTGGAAAATGGCCGCTTTTCTGGATTCATCGACTGTGGCCGGCTGGGTGTGGCGGACCGCTATCAGGACATAGCGTTGGCTACCCGTGATATTGCTGAAGAGCTTGGCGGCGAATGGGCTGACCGCTTCCTCGTGCTTTACGGTATCGCCGCTCCCGATTCGCAGCGCATCGCCTTCTATCGCCTTCTTGACGAGTTCTTCtgaCCCggtaccTCAGCGCTAGCGGAGTGTATACTGGCTTACTATGTTGGCACTGATGAGGGTGTCAGTGAAGTGCTTCATGTGGCAGGAGAAAAAAGGCTGCACCGGTGCGTCAGCAGAATATGTGATACAGGATATATTCCGCTTCCTCGCTCACTGACTCGCTACGCTCGGTCGTTCGACTGCGGCGAGCGGAAATGGCTTACGAACGGGGCGGAGATTTCCTGGAAGATGCCAGGAAGATACTTAACAGGGAAGTGAGAGGGCCGCGGCAAAGCCGTTTTTCCATAGGCTCCGCCCCCCTGACAAGCATCACGAAATCTGACGCTCAAATCAGTGGTGGCGAAACCCGACAGGACTATAAAGATACCAGGCGTTTCCCCCTGGCGGCTCCCTCGTGCGCTCTCCTGTTCCTGCCTTTCGGTTTACCGGTGTCATTCCGCTGTTATGGCCGCGTTTGTCTCATTCCACGCCTGACACTCAGTTCCGGGTAGGCAGTTCGCTCCAAGCTGGACTGTATGCACGAACCCCCCGTTCAGTCCGACCGCTGCGCCTTATCCGGTAACTATCGTCTTGAGTCCAACCCGGAAAGACATGCAAAAGCACCACTGGCAGCAGCCACTGGTAATTGATTTAGAGGAGTTAGTCTTGAAGTCATGCGCCGGTTAAGGCTAAACTGAAAGGACAAGTTTTGGTGACTGCGCTCCTCCAAGCCAGTTACCTCGGTTCAAAGAGTTGGTAGCTCAGAGAACCTTCGAAAAACCGCCCTGCAAGGCGGTTTTTTCGTTTTCAGAGCAAGAGATTACGCGCAGACCAAAACGATCTCAAGAAGATCATCTTATTAAGGGGTCTGACGctcagtggaacgaaaactcacgTTAAGGGATTTTGGTCATGAGATTATCAAAAAGGATCTTCACCTAGATCCTTTTAAATTAAAAATGAAGTTTTAAATCAATCTAAAGTATATATGAGTAAACTTGGTCTGACAGTTACCAATGCTTAATCAGactAGAGCTTccatccgcttgccctcatctgttacgccggcggtagccggccagcctcgcagagcaggattcccgttgagcaccgccaggtgcgaataagggacagtgaagaaggaacacccgctcgcgggtgggcctacttcacctatcctgcccggctgacgccgttggatacaccaaggaaagtctacacgaaccctttggcaaaatcctgtatatcgtgcgAATTGAtccACCGTGCGGCTGCATGAAATCCTGGCCGGTTTGTCTGATGCCAAGCTGGCGGCCTGGCCGGCCAGCTTGGCCGCTGAAGAAACCGAGCGCCGCCGTCTAAAAAGGTGATGTGTATTTGAGTAAAACAGCTTGCGTCATGCGGTCGCTGCGTATATGATGCGATGAGTAAATAAACAAATACGCAAGGGGAACGCATGAAGGTTATCGCTGTACTTAACCAGAAAGGCGGGTCAGGCAAGACgaccatcgcaacccatctagcCCGCGCCCTGCAACTCGCCGGGGCCGATGTTCTGTTAGTCGATTCCGATCCCCAGGGCAGTGCCCGCGATTGGGCGGCCGTGCGGGAAGATCAACCGCTAACCGTTGTCGGCATCGACCGCCCGACGATTGACCGCGACGTGAAGGCCATCGGCCGGCGCGACTTCGTAGTGATCGACGGAGCGCCCCAGGCGGCGGACTTGGCTGTGTCCGCGATCAAGGCAGCCGACTTCGTGCTGATTCCGGTGCAGCCAAGCCCTTACGACATATGGGCCACCGCCGACCTGGTGGAGCTGGTTAAGCAGCGCATTGAGGTCACGGATGGAAGGCTACAAGCGGCCTTTGTCGTGTCGCGGGCGATCAAAGGCACGCGCATCGGCGGTGAGGTTGCCGAGGCGCTGGCCGGGTACGAGCTGCCCATTCTTGAGTCCCGTATCACGCAGCGCGTGAGCTACCCAGGCACTGCCGCCGCCGGCACAACCGTTCTTGAATCAGAACCCGAGGGCGACGCTGCCCGCGAGGTCCAGGCGCTGGCCGCTGAAATTAAATCAAAACTCATTTGAGTTAATGAGGTAAAGAGAAAATGAGCAAAAGCACAAACACGCTAAGTGCCGGCCGTCCGAGCGCACGCAGCAGCAAGGCTGCAACGTTGGCCAGCCTGGCAGACACGCCAGCCATGAAGCGGGTCAACTTTCAGTTGCCGGCGGAGGATCACACCAAGCTGAAGATGTACGCGGTACGCCAAGGCAAGACCATTACCGAGCTGCTATCTGAATACATCGCGCAGCTACCAGAGTAAATGAGCAAATGAATAAATGAGTAGATGAATTTTAGCGGCTAAAGGAGGCGGCATGGAAAATCAAGAACAACCAGGCACCGACGCCGTGGAATGCCCCATGTGTGGAGGAACGGGCGGTTGGCCAGGCGTAAGCGGCTGGGTTGTCTGCCGGCCCTGCAATGGCACTGGAACCCCCAAGCCCGAGGAATCGGCGTGACGGTCGCAAACCATCCGGCCCGGTACAAATCGGCGCGGCGCTGGGTGATGACCTGGTGGAGAAGTTGAAGGCCGCGCAGGCCGCCCAGCGGCAACGCATCGAGGCAGAAGCACGCCCCGGTGAATCGTGGCAAGCGGCCGCTGATCGAATCCGCAAAGAATCCCGGCAACCGCCGGCAGCCGGTGCGCCGTCGATTAGGAAGCCGCCCAAGGGCGACGAGCAACCAGATTTTTTCGTTCCGATGCTCTATGACGTGGGCACCCGCGATAGTCGCAGCATCATGGACGTGGCCGTTTTCCGTCTGTCGAAGCGTGACCGACGAGCTGGCGAGGTGATCCGCTACGAGCTTCCAGACGGGCACGTAGAGGTTTCCGCAGGGCCGGCCGGCATGGCCAGTGTGTGGGATTACGACCTGGTACTGATGGCGGTTTCCCATCTAACCGAATCCATGAACCGATACCGGGAAGGGAAGGGAGACAAGCCCGGCCGCGTGTTCCGTCCACACGTTGCGGACGTACTCAAGTTCTGCCGGCGAGCCGATGGCGGAAAGCAGAAAGACGACCTGGTAGAAACCTGCATTCGGTTAAACACCACGCACGTTGCCATGCAGCGTACGAAGAAGGCCAAGAACGGCCGCCTGGTGACGGTATCCGAGGGTGAAGCCTTGATTAGCCGCTACAAGATCGTAAAGAGCGAAACCGGGCGGCCGGAGTACATCGAGATCGAGCTAGCTGATTGGATGTACCGCGAGATCACAGAAGGCAAGAACCCGGACGTGCTGACGGTTCACCCCGATTACTTTTTGATCGATCCCGGCATCGGCCGTTTTCTCTACCGCCTGGCACGCCGCGCCGCAGGCAAGGCAGAAGCCAGATGGTTGTTCAAGACGATCTACGAACGCAGTGGCAGCGCCGGAGAGTTCAAGAAGTTCTGTTTCACCGTGCGCAAGCTGATCGGGTCAAATGACCTGCCGGAGTACGATTTGAAGGAGGAGGCGGGGCAGGCTGGCCCGATCCTAGTCATGCGCTACCGCAACCTGATCGAGGGCGAAGCATCCGCCGGTTCCTAATGTACGGAGCAGATGCTAGGGCAAATTGCCCTAGCAGGGGAAAAAGGTCGAAAAGGTCTCTTTCCTGTGGATAGCACGTACATTGGGAACCCAAAGCCGTACATTGGGAACCGGAACCCGTACATTGGGAACCCAAAGCCGTACATTGGGAACCGGTCACACATGTAAGTGACTGATATAAAAGAGAAAAAAGGCGATTTTTCCGCCTAAAACTCTTTAAAACTTATTAAAACTCTTAAAACCCGCCTGGCCTGTGCATAACTGTCTGGCCAGCGCACAGCCGAAGAGCTGCAAAAAGCGCCTACCCTTCGGTCGCTGCGCTCCCTACGCCCCGCCGCTTCGCGTCGGCCTATCGCGGCCGCTGGCCGCTCAAAAATGGCTGGCCTacggccaggcaatctaccagGGCGCGGACAAGCCGCGCCGTCGCCACTCGACCGCCGGCGCCCACATCAAGGCACCCTGCCT

pPG159 insert (SacI – P*hrrP* - EcoRI):

gagctcTCTCGTCGAGAACGTAACCGTCTTGGTCAACGGCACGCAAGAGCCAATGTTTGCGGCCGCCGATGGAAATCACCACCTCGTCCAGATGCCAGATATCCTTTCGCGAAGGCCTCTTTCTGCACAACTGCCTGGCATAAGCCGTCCCGAATTTGCGACCCCATCTCCGGATCGTCTCATGGGAGACGACGATACCGCGCTCCAGCAGCATTTCCTCGACCATCCTTAGGCTCAAAGGGAACCGAAAATACAGCCACACCGCACGGGCGATGATCTGCGGTGGAAAGCGGTGGTTCTTGTAGCTTACGGCCGGACTGTTCATCCCAACCCGTTATCCACAATCGTTAAGCCGCAGACAACGTGACATCGCCATGTCAACGTTTCGTCTCGGTTCACGGCCAGATCGCCAACCTCTTTCATCTCCATCGGAAACATCTCACCGCCAACGATCATCGCCAACTCCGCGCCCAAGCCGTCACCACCTGGCGTGAAATCGCATTGTCGATTGACGCCTGAAAAGCCGACGGTACCACCCTTCAGATCCTGGCTTCCAATAAGGCGACGCCACCGCGGGAGCCTATCGGTCCGGATGACGGCTTGACCAACTCGAATCTTTATCTTACATGACGGCATAACTACGACATAACGCGGATATTCCGTCgaattc

pPG161 insert (EcoRI – P*sapA* - XhoI):

gaattcAATCGGCGTTTTCGTGGCAACCAAGCACGAGAAGAACTCCGCCCCGATGGTCACCCTTGCGACTGCCCATCCGGCAAAATTTCCCGCCGCAGTAAAATCGGCAAGTGGTATTGACCCCACGCTTCCGACGTGGCTTGCTGATCTCATGACTAGGGCGGAGCGTTTCGACATCCTGGATCCGGAGCTCAAGAGCGTCGAAACCTTCATCGGCGAGCGTACCCGCGTTCGGGAATAGAGCGAAAGATCACGGctcgag

pPG168 insert (SacI – P*trp* - EcoRI):

gagctcGGGATGTGCTGCAAGGCGATTAAGTTGGGTAACGCCAGGGTTTTCCCAGTCACGACGTTGTAAAACGACGGCCAGTGCCAAGCTTGGCAAATATACTGAAATAGGTGttgacaTTATTCCATCGAACTAGttaactAGTACGAAAGTTCACATGAAGAGGGTATCTAAAATGGCAGCGACATTTGCATTACACGGTTGGTGGCGCACTTCCTGATAgaattc
